# Leveraging PGPR-enriched biochar for enhanced canola growth in heavy metal-contaminated soil

**DOI:** 10.1186/s12870-026-09582-8

**Published:** 2026-07-21

**Authors:** Tarek Alshaal, Khadiga Alharbi, Alaa El-Dein Omara, Emad M. Hafez, Nevien Elhawat

**Affiliations:** 1 Department of Applied Plant Biology, Institute of Crop Sciences, University of Debrecen, Debrecen, 4032 Hungary; 2 Department of Food Biotechnology, Albert Kazmer Mosonmagyarovar Faculty, Széchenyi István University, Egyetem Sqr. 1, Győr, 9026 Hungary; 3 Soil and Water Department, Faculty of Agriculture, University of Kafrelsheikh, Kafr El- Sheikh, 33516 Egypt; 4 Department of Biology, College of science, Princess Nourah bint Abdulrahman University, P.O.Box 84428, Riyadh, 11671 Saudi Arabia; 5 Department of Microbiology, Water Environment Research Institute, Agricultural Research Center, Soils, Giza, 12112 Egypt; 6 Department of Agronomy, Faculty of Agriculture, Kafrelsheikh University, Kafr El-Sheikh, 33516 Egypt; 7 Institute of Agricultural Resources and Environment, Jiangsu Academy of Agricultural Sciences, Nanjing, 210014 China; 8 Key Laboratory of Agro-Environment downstream of Yangze Plain, Ministry of Agriculture and Rural Affairs of the People’s Republic of China, Nanjing, 210014 China; 9 Faculty of Agriculture (for Girls), Al-Azhar University, Nasr City, 11884 Cairo Egypt

**Keywords:** Soil remediation, Rhizosphere microbiology, Soil enzymatic activity, Oxidative stress tolerance, Crop productivity, Food safety

## Abstract

The reuse of polluted drainage water for irrigation is increasingly unavoidable in arid and semi-arid regions, yet it poses serious risks due to the accumulation of toxic heavy metals in soils and crops. Although biochar and plant growth-promoting rhizobacteria (PGPR) have individually shown potential to alleviate metal stress, field-scale evidence elucidating their synergistic and mechanistic effects under realistic, combined soil- and irrigation-derived contamination remains limited. This study addresses this gap by evaluating the effectiveness of PGPR-enriched biochar in mitigating lead (Pb), cadmium (Cd), and nickel (Ni) stress in canola (*Brassica napus* L.) grown under open-field conditions. A naturally contaminated clay soil was continuously irrigated with polluted drainage water from the Kitchener drain (Egypt), creating chronic heavy metal stress. Biochar was applied at 5 and 10 ton ha⁻¹, alone or enriched with defined PGPR consortia composed of *Bacillus circulans* NCAIM B.02324, *Azospirillum brasiliense* SARS 1001, and *Pseudomonas koreensis* MG209738, applied via seed inoculation. The combined application of 10 ton ha⁻¹ biochar with the three-strain consortium (10BC+PGPR3) produced the strongest responses. This treatment (10BC+PGPR3) significantly enhanced soil microbial respiration and key enzyme activities, indicating improved soil biological functioning, while reducing extractable Pb, Cd, and Ni by 55–65% relative to the untreated control. These soil-level improvements translated into marked reductions in metal uptake and translocation to shoots and seeds, alongside enhanced plant water status, membrane stability, and oxidative stress tolerance. Consequently, seed yield and oil content increased by ~ 60% and ~ 90%, respectively. Overall, this study demonstrates that PGPR-enriched biochar acts through coupled soil biochemical and plant physiological mechanisms to immobilize heavy metals and restore crop productivity under real contaminated irrigation scenarios. The findings provide robust field-based evidence supporting this integrated strategy as a practical and sustainable solution for improving soil health, crop performance, and food safety in heavy metal-affected agroecosystems.

## Introduction

Heavy metal contamination of agricultural soils has become a major environmental and agronomic concern worldwide, particularly in regions where intensive human activities overlap with crop production systems [1, 2]. Toxic elements such as lead (Pb), cadmium (Cd), and nickel (Ni) are of particular concern because of their persistence, non-biodegradability, and tendency to accumulate in soils and plants over time [3]. Repeated inputs from anthropogenic sources, including contaminated irrigation water, industrial effluents, urban runoff, and agrochemical use, result in chronic soil contamination that poses long-term risks to soil health, crop productivity, and food safety [4]. Oilseed crops such as canola (*Brassica napus* L.) are especially vulnerable due to their sensitivity to trace metal stress and their capacity to accumulate metals in edible tissues, raising concerns for both yield stability and human health [5–7].

Excessive concentrations of Pb, Cd, and Ni disrupt soil biochemical functioning by inhibiting microbial growth, suppressing enzymatic activities, and altering nutrient cycling processes in the rhizosphere [8]. These soil-level constraints directly affect plant performance by reducing nutrient availability and increasing the phytoavailability of toxic metals. At the plant level, heavy metals impair photosynthesis, disturb water relations, and interfere with essential ion uptake, while simultaneously inducing oxidative stress through the overproduction of reactive oxygen species (ROS) [9]. The resulting oxidative damage to lipids, proteins, and nucleic acids ultimately manifests as reduced biomass, lower seed yield, and inferior product quality, including contamination of edible plant parts with toxic metals [10, 11].

Traditional remediation approaches, such as soil excavation, chemical amendments, or phytoextraction, are often economically prohibitive, environmentally disruptive, or insufficiently effective under field conditions [12]. Consequently, sustainable and soil-based strategies that simultaneously reduce metal bioavailability and enhance crop resilience have gained increasing attention. Among these, biochar and plant growth-promoting rhizobacteria (PGPR) have emerged as promising tools for mitigating heavy metal stress in agroecosystems [13].

Biochar, a carbon-rich material produced through the pyrolysis of biomass, is characterized by a high surface area, porous structure, and abundant functional groups capable of adsorbing and immobilizing metal ions [3, 14]. Numerous studies have demonstrated that biochar application can reduce the bioavailability of Pb, Cd, and Ni, improve soil structure, enhance water retention, and promote microbial activity, thereby indirectly supporting plant growth in contaminated soils [15]. However, the effectiveness of biochar alone may be constrained by soil type, contamination level, and limited biological interaction with plant roots.

PGPR offer a complementary biological approach to heavy metal stress mitigation. These beneficial microorganisms promote plant growth through multiple mechanisms, including phytohormone production, nutrient solubilization, biological nitrogen fixation, and modulation of plant stress responses [15–17]. Under heavy metal stress, PGPR can secrete siderophores, organic acids, and exopolysaccharides that chelate metals and reduce their mobility in the rhizosphere, while also enhancing plant antioxidant defenses and reducing oxidative damage [3]. Nevertheless, the practical application of PGPR in contaminated soils is often limited by poor survival and inconsistent performance under harsh field conditions.

Recent research indicates that combining biochar with PGPR may overcome these limitations by creating a synergistic system in which biochar acts both as a metal immobilizing agent and as a protective carrier that enhances microbial survival and activity [18]. Biochar-amended soils provide favorable microhabitats that buffer environmental stress, allowing PGPR to colonize the rhizosphere more effectively and exert sustained beneficial effects. Despite growing interest in this integrated approach, most existing studies remain pot-based or short-term, and mechanistic field-scale evidence under realistic multi-metal contamination scenarios remains scarce [19].

Furthermore, many studies focus on either plant growth or metal uptake in isolation, without integrating soil biochemical indicators, plant physiological responses, and yield-level outcomes into a unified framework. In particular, the interactive effects of multi-strain PGPR consortia combined with biochar on soil enzyme activities, microbial respiration, oxidative stress mitigation, and metal translocation to seeds remain poorly understood under open-field conditions [20].

In this context, the present study was conducted under open-field conditions in a naturally contaminated clay soil exposed to Pb, Cd, and Ni, representing a realistic and chronic stress environment rather than artificially induced contamination. Biochar was applied at two rates and enriched with defined PGPR consortia composed of Bacillus circulans, *Azospirillum brasiliense*, and *Pseudomonas koreensis*, selected for their metal tolerance and plant growth-promoting traits [18, 20]. This experimental design allows for a comprehensive evaluation of biological, physiological, and agronomic responses within an integrated soil–plant system.

We hypothesized that PGPR-enriched biochar would synergistically reduce heavy metal bioavailability, enhance soil biological functioning, and strengthen plant physiological resilience, resulting in improved yield and safer canola production under heavy metal stress. Accordingly, the objectives of this study were to: (i) assess changes in soil microbial respiration and key enzyme activities as indicators of soil health; (ii) quantify reductions in extractable Pb, Cd, and Ni and their accumulation and translocation within plant tissues; (iii) evaluate plant physiological, biochemical, and antioxidant responses to combined biochar and PGPR application; and (iv) determine the resulting effects on yield and oil quality of canola.

## Materials and methods

### Experimental site

In 2022, experiments were established in an open field in Kafr El-Sheikh Governorate (31.40° N, 31.17° E), Egypt. The primary source of irrigation water for crops in this area is the Kitchener drain that receives a mixture of industrial water (2%), domestic water (23%), and agricultural drainage water (75%) [21]. Water in the Kitchener drain has the following properties: pH 7.27 ± 0.02; electrical conductivity 0.54 ± 0.01 dS/m; sodium adsorption ratio (SAR) 1.47 ± 0.03; ions concentration (mg/L): Na^+^ 2.01 ± 0.04; Cl^−^ 3.43 ± 0.04; SO_4_^2−^ 0.14 ± 0.02; NH_4_^+^ 1.73 ± 0.02; heavy metal concentration (mg/L): Cd 0.10 ± 0.02; Pb 0.85 ± 0.06; and Ni 0.20 ± 0.04. The permissible concentrations of Pb, Cd, and Ni in water can differ according to the guidelines set by various countries and organizations. For example, the permissible levels for Cd and Ni are 0.01 mg/L and 0.2 mg/L, respectively [22–24]. For Pb, the permissible concentrations are 0.1 mg/L [22], 0.5 mg/L [24], and 0.1 mg/L [23]. As a result, the irrigation water utilized in this study is categorized as polluted with Pb, Cd, and Ni.

The soil used in this study is classified as clayey which falls under the order Vertisols based on the United States Department of Agriculture (USDA) Soil Taxonomy system, with the following physicochemical characteristics: pH, 8.20 ± 0.01 (in 1:2.5 soil: water suspension); electrical conductivity (in soil paste extract), 4.64 ± 0.02 dS/m; SOM (soil organic matter), 11.1 ± 0.02 g/kg; ESP (exchangeable sodium percentage), 22.4 ± 0.40%; cations and anions (meq/L) Ca^2+^, 7.55 ± 0.91; Mg^2+^, 5.78 ± 1.10; Na^+^, 26.73 ± 2.01; K^+^, 0.35 ± 0.01; HCO_3_^−^, 4.63 ± 0.53; Cl^−^, 24.58 ± 1.10; SO_4_^2−^, 15.15 ± 3.01; available N, 9.73 ± 0.90 mg/kg; available P, 8.26 ± 1.31 mg/kg; available K, 346 ± 26.40 mg/kg; total Cd, 2.05 ± 0.18 mg/kg; and extractable Cd, 0.81 ± 0.04 mg/kg; total Pb, 31.28 ± 1.81 mg/kg; and extractable Pb, 6.17 ± 0.14 mg/kg; total Ni, 48.69 ± 2.82 mg/kg; and extractable Ni, 9.58 ± 0.24 mg/kg. Compared to WHO and FAO guidelines for agricultural soils (Pb: 50 mg/kg, Cd: 3 mg/kg, Ni: 50 mg/kg; [22, 24]), the soil exhibits moderate to high contamination, particularly in bioavailable fractions, posing risks for crop uptake. The primary irrigation source, the Kitchener drain, contains Pb (0.85 ± 0.06 mg/L), Cd (0.10 ± 0.02 mg/L), and Ni (0.20 ± 0.04 mg/L), exceeding permissible limits (Pb: 0.1–0.5 mg/L, Cd: 0.01 mg/L, Ni: 0.2 mg/L; [22–24]), further contributing to soil contamination. Soil samples were collected from a depth of 0–30 cm before the trial began.

### Treatments and experimental layout

The experiment involved two biochar application rates (5 and 10 tons/ha), each applied either alone or combined with three types of PGPR consortia, and incorporated thoroughly into the top 30 cm of soil:: PGPR1 (*Bacillus circulans* NCAIM B.02324 + *Azospirillum brasiliense* SARS 1001), PGPR2 (*Pseudomonas koreensis* MG209738 + *Azospirillum brasiliense* SARS 1001), and PGPR3 (*Bacillus circulans* NCAIM B.02324 + *Azospirillum brasiliense* SARS 1001 + *Pseudomonas koreensis* MG209738). Additionally, a negative control (CK) with no BC and no PGPR inoculation was included, resulting in a total of 12 treatments with four replicates each: CK (negative control); PGPR1 (*Bacillus circulans* NCAIM B.02324 + *Azospirillum brasiliense* SARS 1001); PGPR2 (*Pseudomonas koreensis* MG209738 + *Azospirillum brasiliense* SARS 1001); PGPR3 (*Bacillus circulans* NCAIM B.02324 + *Azospirillum brasiliense* SARS 1001 + *Pseudomonas koreensis* MG209738); 5BC (5 ton/ha biochar); 5BC+PGPR1 (5 ton/ha biochar+PGPR1); 5BC+PGPR2 (5 ton/ha biochar+PGPR2); 5BC+PGPR3 (5 ton/ha biochar+PGPR3); 10BC (10 ton/ha biochar); 10BC+PGPR1 (10 ton/ha biochar+PGPR1); 10BC+PGPR2 (10 ton/ha biochar+PGPR2); and 10BC+PGPR3 (10 ton/ha biochar+PGPR3). The three PGPR consortia (PGPR1, PGPR2, and PGPR3) were designed to progressively combine strains with complementary and non-redundant plant growth–promoting traits, rather than simply increasing the number of strains. The experimental design followed a Factorial Completely Randomized layout, with BC as the main plots and PGPRs as subplots. Each experimental plot consisted of 6 rows with 50 cm line spacing, covering an area of 10.5 m².

### Biochar properties

Biochar was produced through the pyrolysis of a 1:1 mixture of corn stalk and rice husk [15]. The properties of the resulting BC were as follows: pH 7.62 ± 0.01 (1:5, BC: water suspension); electrical conductivity 0.71 ± 0.01 dS/m (1:5, BC: water extract); CaCO_3_ content 1.5 ± 0.01%; density 0.20 ± 0.01 g/cm³; specific surface area 37.2 ± 2.11 m²/g; water-binding capacity 35.05 ± 2.21%; water content 11.6 ± 0.99%; nitrogen (N) content 25.25 ± 2.93 mg/kg; phosphorus (P) content 7.47 ± 0.81 mg/kg; potassium (K) content 13.23 ± 1.41 mg/kg.

### PGPR inocula

*Azospirillum brasiliense* SARS 1001, *Bacillus circulans* NCAIM B.02324, and *Pseudomonas koreensis* MG209738 were provided by the Agricultural Research Center, Giza, Egypt. The semi-solid malate medium was applied to culture the *(A) brasiliense* SARS 1001 strain [25], while the *(B) circulans* NCAIM B.02324 strain was cultivated on the nutrient broth medium [26], and the King’s B (KB) broth medium was used to grow the *P. koreensis* MG209738 strain. The bacterial inoculum was prepared by loading 15 mL of bacterial suspension (10^8^ CFU/mL) onto 30 g of pasteurized peat carrier. The rate at which canola seeds were treated before sowing was 950 g inoculum/ ha and was carried out under shaded conditions.

### Experimental description

The canola (*Brassica napus* L., cv. Misr 1) seeds were provided by the Agricultural Research Center, Giza, Egypt. Canola was selected due to its high agronomic and economic importance as a major global oilseed crop used for edible oil, biodiesel, and protein-rich meal. It is moderately sensitive to heavy metals such as Cd and Pb, which can accumulate in seeds, making it a suitable indicator for assessing strategies that reduce metal bioavailability and ensure food safety. Additionally, canola exhibits strong physiological and biochemical responses to soil amendments, enabling mechanistic evaluation of stress mitigation by biochar and PGPR within a phytomanagement framework [7, 27–29]. Sowing took place on October 21, 2022, at a rate of 6 kg/ha. The growing season lasted for approximately seven months. Seeds were planted in hills, with 3–4 seeds per hill, and spaced 10 cm apart on a single ridge. Fifteen days after sowing, the germinated seeds were thinned to two plants per hill.

Surface irrigation was carried out every 21 days using water from the Kitchener drain. The experimental field was plowed twice before sowing the seeds. During the second plowing, calcium superphosphate (15.5%) was incorporated into the soil at 470 kg/ha. Ammonium nitrate (33.5% N) was applied at 350 kg N/ha and divided into two equal applications during the first two irrigations. Potassium sulfate (48%) was also applied at a rate of 120 kg/ha alongside the second nitrogen application. Stomp herbicide (3 L/ha) was used for weed control.

### Soil measurements

Soil samples were collected at 80 days (for biological and biochemical analyses) and 120 days (for chemical analysis) after seed sowing, from a depth of 0–20 cm in each experimental plot, with three replicates. Plant remains, stones, and gravels were removed, and soil samples were then air-dried at the lab conditions. The three replicates were well-mixed to obtain one representative soil sample. Before being stored in plastic bags, soil samples were passed through a 2 mm sieve. For soil biological and biochemical parameters, soil samples were gothered in sterilized polyethylene bags and transported to the laboratory in an icebox. Three surface soil samples (0–20 cm) per plot were admixed to have one combined sample per plot. These samples were screened to remove plant material, stones, or gravel, sieved through an 8 mm sieve, and stored at -20 °C for future analyses. Soil pH (in 1:2.5 soil: water suspension) was measured by a pH meter (Genway 3510, Cambridgeshire, UK), while electrical conductivity (EC_e_) was determined by an EC meter (Jenway 4310, Genway, Cambridgeshire, UK) using soil paste extract [30]. Extractable soil Cd, Pb, and Ni were extracted using EDTA and measured by Atomic Absorption Spectrophotometry (AAS, PerkinElmer 3300, Shelton, USA) [31]. Glucose-induced soil respiration was measured in thawed soil samples at room temperature for 24 h using NaOH after 10-day incubation at 35 °C, following the addition of 80 mg glucose/g soil [32]. Soil moisture content in the samples was adjusted to 60%. Soil dehydrogenase activity was measured spectrophotometrically using the 2,3,5-triphenyl tetrazolium chloride (TTC) method (3% w/v) as described by [33], with absorbance recorded at 485 nm and a standard curve generated using triphenyl formazan (TPF), after incubation samples at 37 °C for 24 h. Soil phosphatase activity was assessed using p-nitrophenyl phosphate as a substrate, with absorbance measured at 440 nm, after incubation at 37 °C for 1 h [34]. Soil urease activity was analyzed using urea as the substrate, and measured spectrophotometrically at 670 nm, after incubation at 37 °C for 24 h [35].

### Plant water status and membrane stability

Eighty days after seed sowing, the youngest fully developed leaves from the plant tips were collected for the determination of physiological and biochemical markers of stress. The relative water content (RWC) was determined using 1 cm² leaf discs. Initially, the fresh mass (FM) of the discs was recorded. The discs were then soaked in distilled water for 5 h to obtain the turgid mass (TM). Following this, the discs were dried at 70 °C for 48 h, and the dry mass (DM) was recorded. The RWC was calculated using the formula provided by [36].

$$\:RWC\:\left(\%\right)=\frac{FM-DM\:}{TM-DM}\times\:100$$

To assess electrolyte leakage (EL), ten leaf discs (each with an area of 1 cm²) were placed in a test tube containing 10 mL of distilled water and heated in a water bath at 55 °C for 25 min. The electrical conductivity (EC1) was then measured. Subsequently, the samples were heated to 100 °C for an additional 10 min to obtain a second electrical conductivity measurement (EC2). The EL was calculated using the method outlined by [37].

$$\:EL\:\left(\%\right)=\frac{EC1}{EC2}\:\times\:100$$

The malondialdehyde (MDA) content, which serves as a marker of lipid peroxidation in the phospholipid bilayer, was determined from 0.5 g of fresh leaves that were ground in liquid nitrogen. The thiobarbituric acid (TBA) method, as described by [38], was employed. The absorbance of the resulting solution was measured at wavelengths of 532 nm and 600 nm using a UV-160 A spectrophotometer (Shimadzu, Kyoto, Japan).The hydrogen peroxide (H₂O₂) content was quantified from 1 g of fresh leaves that were ground in liquid nitrogen using the trichloroacetic acid (TCA) method as outlined by [39]. The absorbance of the resulting yellow supernatant was measured at 426 nm using a UV-160 A spectrophotometer (Shimadzu, Kyoto, Japan).

### Oxidative stress indicators and antioxidant defense mechanisms

#### Determination of non-enzymatic antioxidant systems

Soluble protein content was quantified in a 20 mg lyophilized leaf sample after homogenization using the Coomassie Brilliant Blue G-250 method, following Bradford (1976). The absorbance of the generated blue color was recorded at 595 nm using a UV-160 A spectrophotometer (Shimadzu, Kyoto, Japan). Bovine serum albumin was used to generate the standard curve.

Total soluble sugars (TSS) were extracted from 0.5 g of fresh leaves using 80% hot ethanol, following the anthrone method described by [40]. The absorbance of the resulting supernatant was measured at 625 nm, and the TSS concentration was determined using a glucose standard curve. Glycine betaine (GB) content was extracted from 0.5 g of fresh leaves using deionized water at 25 °C for 24 h, following the method outlined by [41]. The filtrate was subsequently diluted with 1 M H₂SO₄ and mixed with a chilled KI-I₂ reagent. The absorbance was measured at 365 nm, and the GB concentration was determined using a GB standard curve. 500 mg fresh leaves were ground in 3% H_2_SO_4_ to determine proline content, followed by centrifugation at 12,000 rpm for 5 min. The supernatant was mixed with toluene, and the proline content was quantified using ninhydrin reagent according to the method of [42]. The absorbance of the resulting color was measured at 520 nm using a UV-160 A spectrophotometer (Shimadzu, Kyoto, Japan). Total contents of polyphenols (TPC) and flavonoids (TFC) were extracted from the youngest fully expanded canola leaves using a methanol: distilled water solution (70:30). A 20 mg lyophilized sample was placed in a 1.5 mL Eppendorf tube, and 1 mL of the extraction solution was added. The mixtures were vortexed for 2 min at room temperature and then incubated in an ultrasonic bath for 60 min at room temperature. After centrifugation at 10,000 rpm for 5 min at room temperature, the supernatants were collected. TPC in the supernatants was determined using the Folin-Ciocalteu reagent as described by [43], with the absorbance of the resulting blue color measured at 760 nm using a UV-160 A spectrophotometer (Shimadzu, Kyoto, Japan). The TPC concentration was calculated using a gallic acid standard. For TFC quantification, 500 µL of the supernatants was used in an aluminum chloride assay according to the method reported by [44], with the absorbance of the resulting color measured at 415 nm using a UV-160 A spectrophotometer (Shimadzu, Kyoto, Japan). The TFC concentration was calculated using a rutin standard.

#### Determination of antioxidant enzymes

Dehydroascorbate reductase (DHAR) activity in canola leaves at 80 days after planting was assessed following the method outlined by [45]. Briefly, 0.7 g of fresh leaves were homogenized in potassium phosphate buffer (pH 7.8) mixed with 2 mol/L mercaptoethanol, 1 mol/L EDTA, and 8 v/v% glycerol. The supernatants obtained after centrifugation of the homogenate were used to measure DHAR activity by monitoring absorbance at 265 nm. The extinction coefficient of the enzyme’s substrate was determined to be 7.0 mM/cm. Enzyme activity was quantified as nmol dehydroascorbate reduced per minute per gram fresh weight (FW).

Glutathione reductase (GR) activity was measured in 0.7 g of fresh leaves harvested 80 days after seeding, using potassium phosphate buffer (pH 7.8) supplemented with 2 mol/L EDTA, as described by [46]. The reaction mixture contained 0.15 mM NADPH, 0.5 mM GSSG, 2 mM EDTA, 50 mM potassium phosphate buffer, and 0.2 mL of enzyme extract. Absorbance readings were taken at 340 nm, and the extinction coefficient of the enzyme’s substrate was determined to be 6.2 mM/cm. Enzyme activity was quantified as nmol NADPH consumed per minute.

The activity of ascorbate peroxidase (APX) was measured by monitoring the reduction in absorbance of ascorbic acid at 290 nm, using an extinction coefficient of 2.8 mM/cm. The reaction mixture, totaling 1 mL, included 50 mM phosphate buffer (pH 7.6), 0.1 mM Na-EDTA, 12 mM H₂O₂, 0.25 mM ascorbic acid, and the sample extract, according to the method described by [47].

Polyphenol oxidase (PPO) activity was assessed in canola leaves by homogenizing the plant tissue in potassium phosphate buffer (pH 7.8) to extract the enzyme, followed by centrifugation as described by [48]. The reaction mixture contained 50 mM phosphate buffer (pH 7.0) and 20 mM catechol. PPO activity was indicated by the absorbance recorded at 420 nm, which reflects the formation of quinones from catechol oxidation. One unit of PPO activity (U/mL) is defined as the amount of enzyme that results in an increase in absorbance of 0.001 per minute under the assay conditions.

### Photosynthetic parameters

Photosynthetic pigments were assessed 80 days after seed sowing using the method outlined by the method of [49]. In brief, pigments were extracted from 1 g of fresh leaf tissue using 6 mL of 80% acetone. The samples were incubated in the dark at room temperature overnight and then centrifuged at 12,000 rpm for 15 min. The absorbance of the resulting supernatant was measured at 645 nm, 663 nm, and 470 nm using a UV-160 A spectrophotometer (Shimadzu, Kyoto, Japan). The following formulas were applied to calculate the contents of chlorophyll a, chlorophyll b, and carotenoids (mg/g FW):

$$\text{Chlorophyll a}=\:({\mathrm{12.7}}-(\mathrm{A}_\mathrm{663})-\mathrm{2.69}\:(\mathrm{A}_\mathrm{645})$$

$$\text{Chlorophyll b}=\:({\mathrm{25.8}}-(\mathrm{A}_\mathrm{645})-\mathrm{4.68}\:(\mathrm{A}_\mathrm{663})$$

$$\mathrm{Carotenoids}=\:({\mathrm{1000}}\:(\mathrm{A}_\mathrm{470})-\mathrm{2.27}\:(\text{chl a})-\mathrm{81.4}\:(\text{chl b}))\:/\:227$$

The rate of photosynthesis was quantified in the youngest fully expanded leaf after eighty days of seed sowing. The assessment was performed under a light intensity of 1000 µmol photons/m²/s as the optimal conditions using the LI-6400 portable photosynthesis system (Li-COR, Lincoln, NE, USA) at 30 ± 2 °C. The CO₂ concentration ranged from 350 to 400 µmol/mol, and the vapor pressure deficit (VPD) was adjusted to correspond to 50% relative humidity (RH), following the method described by [50].

The LI-6400 portable gas exchange system (Li-COR, Lincoln, NE, USA) was used to measure stomatal conductance, which provides a light intensity (PAR) of ≥ 1200 µmol photons/m²/s, during the period from 12:00 to 14:00 h.

### Determination of cellular Na⁺–K⁺ balance

To determine Na⁺ and K⁺ contents in canola leaves, 0.5 g of ground plant material was placed in a Kjeldahl digestion tube, followed by the addition of 5 mL of sulfuric acid (H₂SO₄, 95–97%, 1.84 kg/L, Merck). The tubes were heated with the temperature gradually increased by 5 °C per minute until reaching 170 °C, and digestion was maintained at this temperature for 2 h. After cooling for 30 min, 2 mL of 30% H₂O₂ were added, and the temperature was raised to 120 °C for an additional hour until the digestion solution became clear. The sample volume was then adjusted to 50 mL in a volumetric flask using ultra-pure water. Na⁺ and K⁺ contents were measured using an Atomic Absorption Spectrophotometer (AAS, Perkin Elmer 3300, Shelton, USA) with a detection limit of 100 ppb [51].

### Collecting and preparation of plant samples at harvest

At the end of the experiment, five plants per plot were harvested randomly. Root, shoot, and seeds of selected plants were grouped individually. To eliminate surface contaminants, the plant samples were washed with distilled water, air-dried, and placed in an air-forced oven at 70 °C for 24 h. Dried samples were powdered using a bead mill (model: EDW-50, Shanghai, China) and kept in plastic bags.

### Determination of Cd, Pb, and Ni contents

Concentrated HNO_3_ and HClO_4_ (3:1 v/v) were used for digesting the powdered plant samples. 0.5 g plant sample was mixed with 9 mL HNO_3_ in the 250-mL Kjeldahl digestion tube then placed on a heater (Tecator Digestion unit, VELP Digester model DK 42/26, VELP Scientifica Ltd., Usmate (MB), Italy). The resulting digested mixture was diluted to a final volume of 25 mL with distilled water and filtered through Whatman filter paper 40. Atomic Absorption Spectrophotometry (PerkinElmer 3300, Shelton, USA) was used to quantify the Cd, Pb, and Ni contents [52].

### Uptake and accumulation patterns of trace metals

Bioconcentration factor (BCF) and translocation factor (TF) were computed as follows [53, 54]:

   

$$\:BCF=\frac{Heavy\:metal\:in\:plant\:root\:\left(\frac{\mu\:g}{g}\right)}{Heavy\:metal\:in\:soil\:\left(\frac{mg}{kg}\right)}$$

  

$$\:TF=\frac{Heavy\:metal\:in\:plant\:shoot\:\left(\frac{\mu\:g}{g}\right)}{Heavy\:metal\:in\:plant\:root\:\left(\frac{\mu\:g}{g}\right)}$$

### Seed and oil yields

At harvest, ten plants were randomly selected from each sub-plot to evaluate yield traits, including plant height, number of pods per plant, and number of branches per plant. To assess seed yield, plants were collected from a one-square-meter area in each treatment. The seeds were manually threshed, dried, and then stored. The weight of 1,000 seeds and the seed yield (kg/ha) were measured at a moisture content of 10%. The oil content of the seeds, based on dry matter, was determined using a Soxhlet extractor following the [55] method. The oil yield (kg/ha) was calculated by multiplying the seed yield (kg/ha) by the oil content.

### Statistical evaluation

Data were analyzed using Microsoft Excel 2016 and SPSS version 25.0 (SPSS Inc., Chicago, IL, USA). The experiment was arranged as a factorial completely randomized design in a split-plot arrangement, where biochar (BC) levels were assigned to main plots and PGPR treatments were allocated to subplots, resulting in 12 treatment combinations. Accordingly, the data were subjected to a split-plot analysis of variance (ANOVA), with BC effects tested against the main-plot error term, while PGPR effects and the BC × PGPR interaction were tested against the subplot error term. When significant differences were detected, Tukey’s honestly significant difference (HSD) test was applied for mean separation among treatment combinations at *p* ≤ 0.05. Results are presented as mean ± standard deviation (SD).

## Results

### Soil biochemical and biological responses

**Fig. 1:**
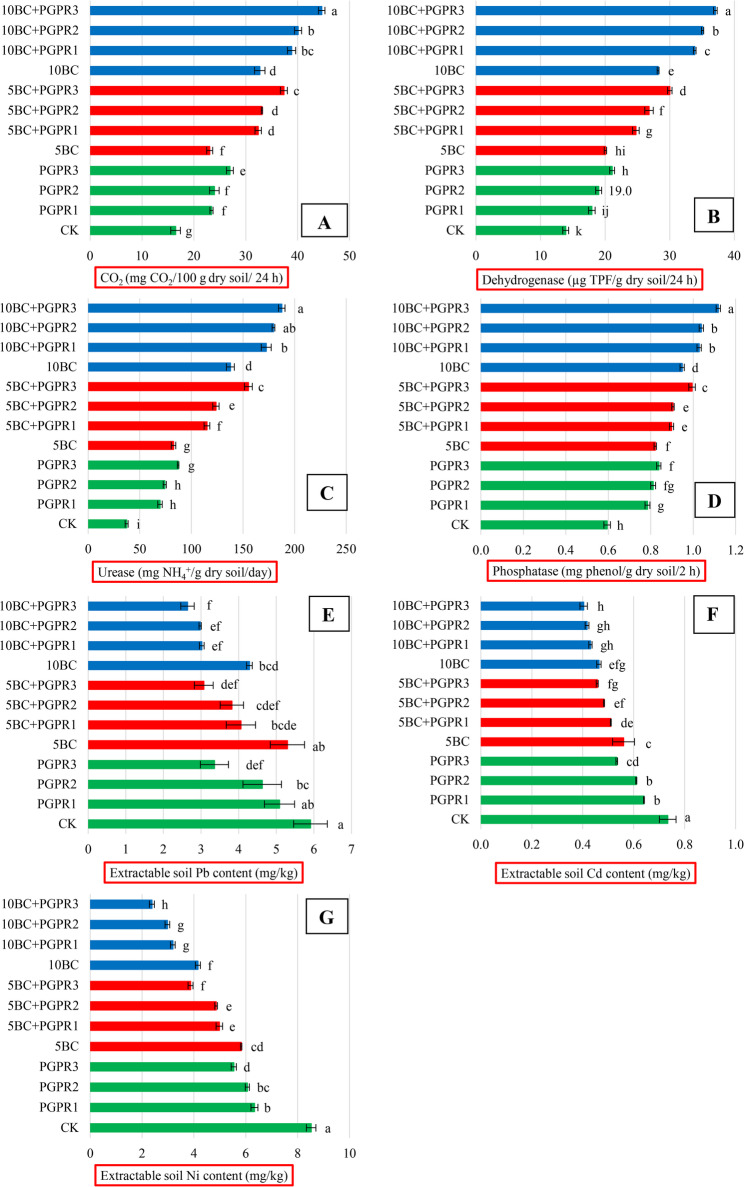
(**A**) Soil respiration, (**B**) dehydrogenase activity, (**C**) urease activity, (**D**) phosphatase activity, (**E**) extractable Pb, (**F**) extractable Cd, and (**G**) extractable Ni contents in soil cultivated with canola (*Brassica napus* L.), which is irrigated with drainage water from the Kitchener drain in contaminated soil with Pb, Cd, and Ni and treating with biochar (5 and 10 ton/ ha BC) which is fortified with three different PGPR consortia, i.e., PGPR1 (*Bacillus circulans* NCAIM B.02324 + *Azospirillum brasiliense* SARS 1001), PGPR2 (*Pseudomonas koreensis* MG209738 + *Azospirillum brasiliense* SARS 1001), and PGPR3 (*Bacillus circulans* NCAIM B.02324 + *Azospirillum brasiliense* SARS 1001 + *Pseudomonas koreensis* MG209738). A negative control was applied (CK). Different letters on bars are significant according to the Tukey’s test (*p* ≤ 0.05). Data are means ± SD (standard deviation) (*n* = 4)

The open-field experiment assessing the effects of BC and PGPR on canola plants grown in Pb, Cd, and Ni-contaminated soil irrigated with similarly contaminated drainage water revealed significant findings (Fig. 1). The control (CK), without BC or PGPR, exhibited the lowest levels of soil respiration (16.5), dehydrogenase (13.9), urease (37.3), and phosphatase (0.59), alongside the highest contents of extractable Pb (5.91 mg/kg), Cd (0.73 mg/kg), and Ni (8.52 mg/kg). PGPR treatments improved soil enzyme activities and reduced heavy metal content in the plants compared to the control. Among the PGPR treatments, PGPR3 showed the highest soil respiration (26.9), highest enzyme activities (dehydrogenase: 21.1, urease: 87.1, phosphatase: 0.84), and the lowest heavy metal contents (Pb: 3.36 mg/kg, Cd: 0.53 mg/kg, Ni: 5.54 mg/kg). BC alone also enhanced soil health and reduced heavy metal uptake. Combined BC and PGPR Treatments: The combination of BC and PGPR yielded the best results: 10BC+PGPR3 demonstrated the highest soil enzyme activities (dehydrogenase: 37.1, urease: 187.5, phosphatase: 1.12), highest soil respiration (44.6), and the lowest heavy metal contents (Pb: 2.64 mg/kg, Cd: 0.40 mg/kg, Ni: 2.38 mg/kg).

### Plant water status and membrane stability

**Fig. 2:**
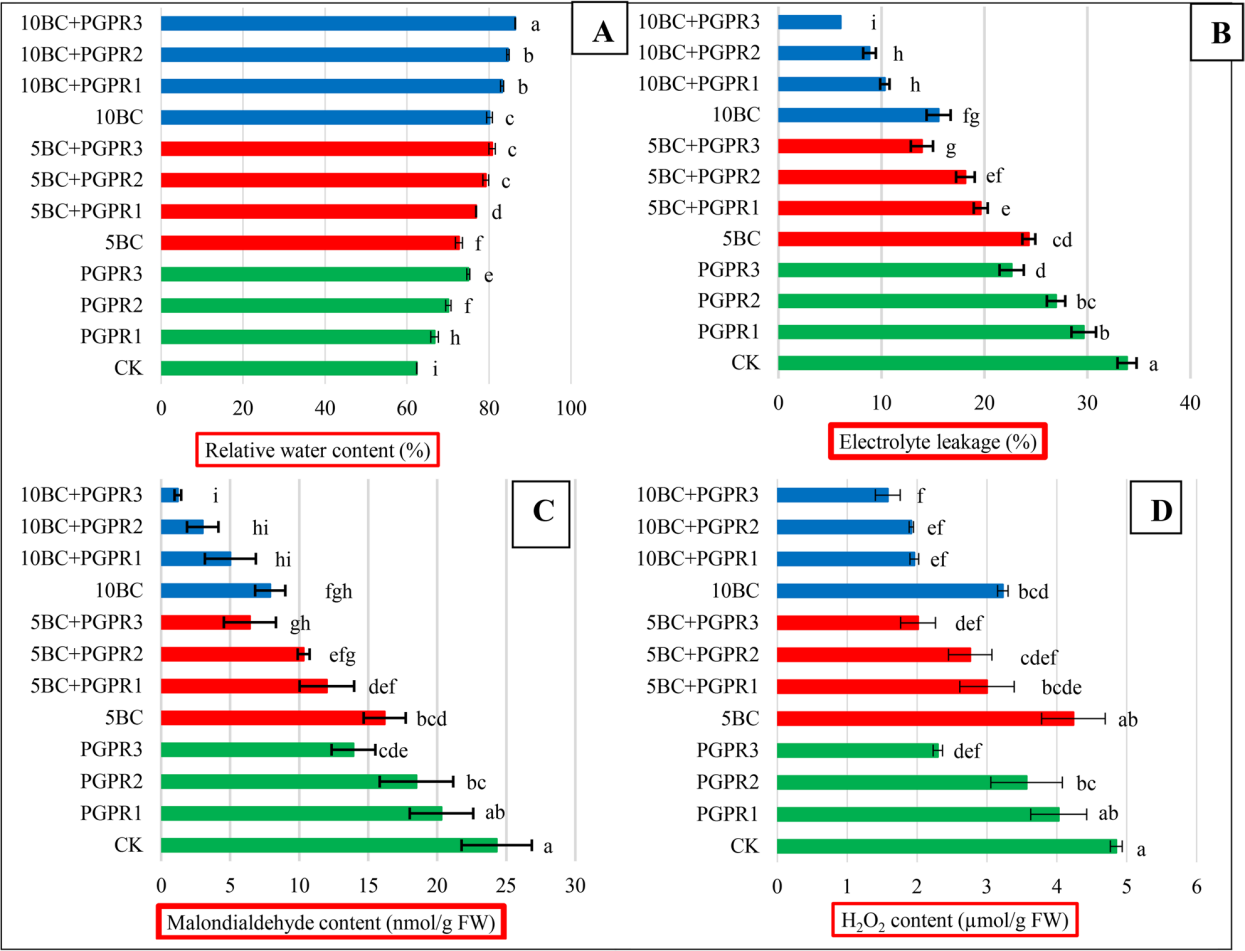
(**A**) Relative water content (RWC), (**B**) electrolyte leakage (EL), (**C**) malondialdehyde (MDA), and (**D**) hydrogen peroxide (H_2_O_2_) contents in leaves of canola (*Brassica napus* L.) irrigated with drainage water from the Kitchener drain in contaminated soil with Pb, Cd, and Ni and treating with biochar (5 and 10 ton/ ha BC) which is fortified with three different PGPR consortia, i.e., PGPR1 (*Bacillus circulans* NCAIM B.02324 + *Azospirillum brasiliense* SARS 1001), PGPR2 (*Pseudomonas koreensis* MG209738 + *Azospirillum brasiliense* SARS 1001), and PGPR3 (*Bacillus circulans* NCAIM B.02324 + *Azospirillum brasiliense* SARS 1001 + *Pseudomonas koreensis* MG209738). A negative control was applied (CK). Different letters on bars are significant according to the Tukey’s test (*p* ≤ 0.05). Data are means ± SD (standard deviation) (*n* = 4)

The CK exhibited the lowest RWC (62.4%) and the highest levels of EL (33.84%), MDA content (24.31 nmol/g FW), and H2O2 content (4.85 µmol/g FW). This highlights the severe stress conditions faced by plants in contaminated soil without any treatment. PGPR1, PGPR2, and PGPR3 treatments progressively improved plant stress indicators compared to the CK. PGPR3 was the most effective among the PGPR treatments, showing significant improvements in RWC (74.9%), reduced EL (22.64%), lower MDA content (13.93 nmol/g FW), and decreased H_2_O_2_ content (2.30 µmol/g FW). The 5 and 10 BC (ton/ha) treatments enhanced plant health compared to the CK. The 10BC showed better results than 5BC, with higher RWC (80.1%), lower EL (15.55%), reduced MDA content (7.90 nmol/g FW), and decreased H_2_O_2_ content (3.23 µmol/g FW). The combination of BC and PGPR showed the best results overall. The 10BC+PGPR3 was the most effective treatment, resulting in the highest RWC (86.4%), the lowest EL (6.04%), minimal MDA content (1.21 nmol/g FW), and the lowest H2O2 content (1.58 µmol/g FW) (Fig. 2).

### Oxidative stress markers and antioxidant defense system

#### Non-enzymatic antioxidants

**Fig. 3:**
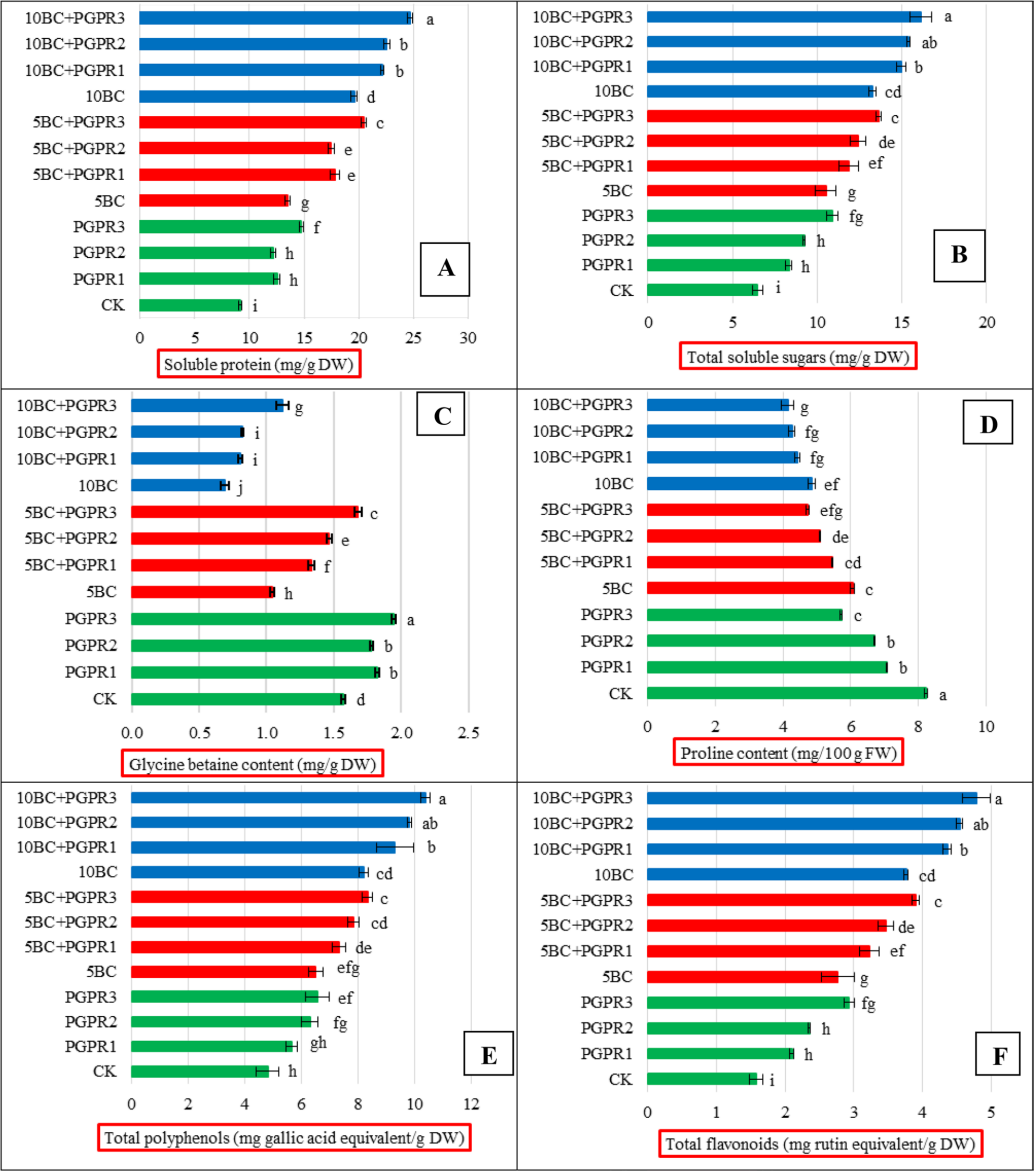
(**A**) Total soluble protein, (**B**) total soluble sugars, (**C**) glycine betaine (GB), (**D**) proline contents, (**E**) total polyphenol, and (**F**) total flavonoid contents in leaves of canola (*Brassica napus* L.) irrigated with drainage water from the Kitchener drain in contaminated soil with Pb, Cd, and Ni and treating with biochar (5 and 10 ton/ ha BC) which is fortified with three different PGPR consortia, i.e., PGPR1 (*Bacillus circulans* NCAIM B.02324 + *Azospirillum brasiliense* SARS 1001), PGPR2 (*Pseudomonas koreensis* MG209738 + *Azospirillum brasiliense* SARS 1001), and PGPR3 (*Bacillus circulans* NCAIM B.02324 + *Azospirillum brasiliense* SARS 1001 + *Pseudomonas koreensis* MG209738). A negative control was applied (CK). Different letters on bars are significant according to the Tukey’s test (*p* ≤ 0.05). Data are means ± SD (standard deviation) (*n* = 4)

The CK plants had the lowest values of soluble protein and TSS, and the highest proline content, indicating high stress levels. All PGPR treatments increased soluble protein and TSS, with PGPR3 being the most effective (Fig. 3). Proline content decreased with all PGPR treatments, indicating reduced stress, with PGPR3 showing the largest reduction. Both 5 and 10 (ton/ha) BC significantly improved soluble protein and TSS, with 10 (ton/ha) BC being more effective. However, glycine betaine content decreased with higher BC concentration, and proline content was lower than the CK, indicating reduced stress. Combining BC with PGPR produced the highest values of soluble protein and TSS. The combination of 10 (ton/ha) BC with PGPR3 (10BC+PGPR3) was the most effective, resulting in the highest levels of soluble protein (24.67 mg/g DW) and TSS (16.11 mg/g DW), and the lowest proline content (4.15 mg/100 g FW). Both TPC and TFC increased significantly with the addition of BC and PGPR compared to the CK. The highest increase was observed in the treatment combining 10 (ton/ha) BC with PGPR3, which recorded TPC of 10.40 and TFC of 4.79.

#### Antioxidant enzymes

**Fig. 4:**
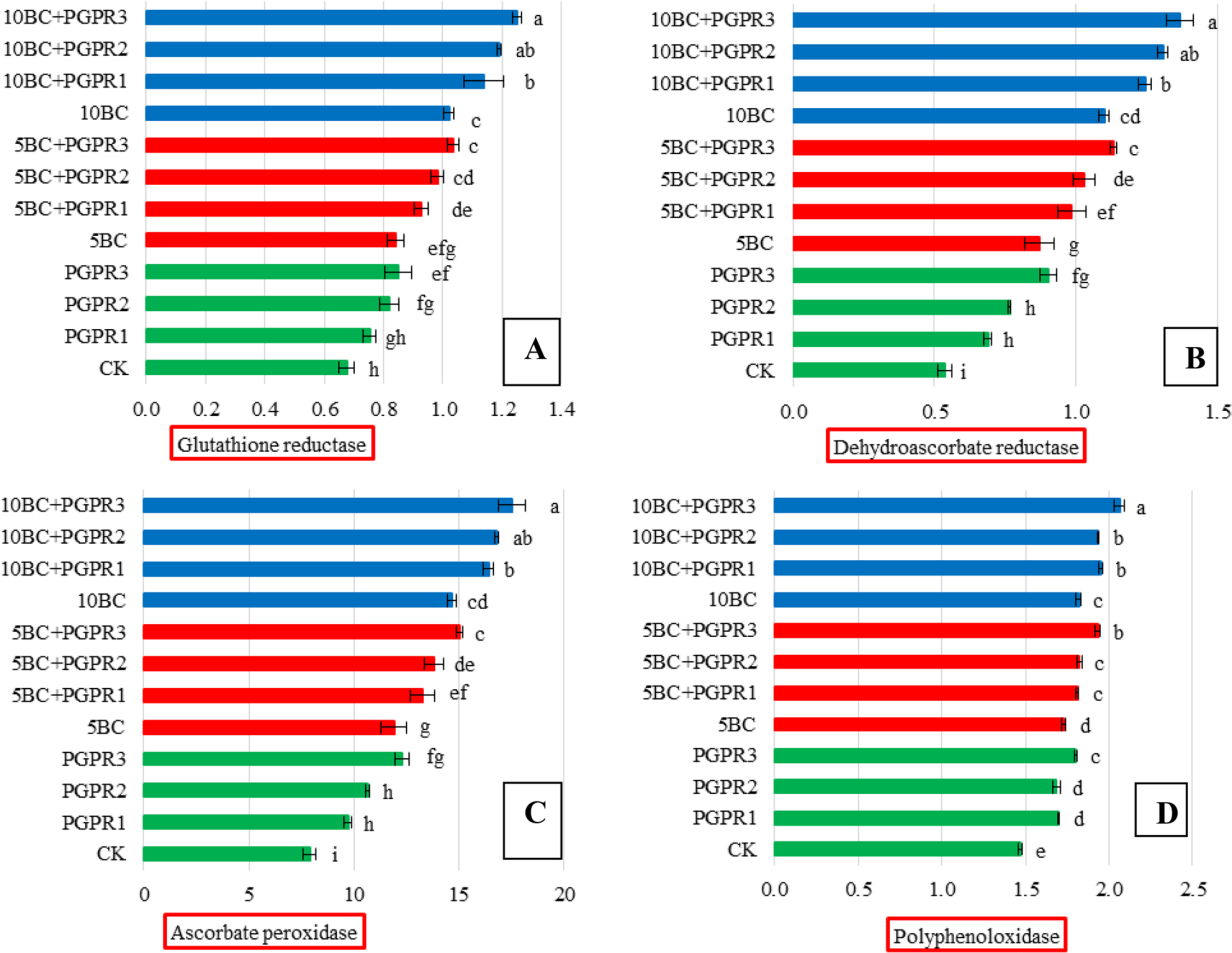
(**A**) Glutathione reductase (GR) activity (nmol NADPH/min), (**B**) dehydroascorbate reductase (DHAR) activity (nmol dehydroascorbate/min/g FW), (**C**) ascorbate peroxidase (APX) activity (µmol ascorbate/min), and (**D**) polyphenoloxidase (PPO) activity (U/mL) in canola (*Brassica napus* L.) irrigated with drainage water from the Kitchener drain in contaminated soil with Pb, Cd, and Ni and treating with biochar (5 and 10 ton/ ha BC) which is fortified with three different PGPR consortia, i.e., PGPR1 (*Bacillus circulans* NCAIM B.02324 + *Azospirillum brasiliense* SARS 1001), PGPR2 (*Pseudomonas koreensis* MG209738 + *Azospirillum brasiliense* SARS 1001), and PGPR3 (*Bacillus circulans* NCAIM B.02324 + *Azospirillum brasiliense* SARS 1001 + *Pseudomonas koreensis* MG209738). A negative control was applied (CK). Different letters on bars are significant according to the Tukey’s test (*p* ≤ 0.05). Data are means ± SD (standard deviation) (*n* = 4)

Antioxidant enzyme activities were compared among the treatments: glutathione reductase (GR), dehydroascorbate reductase (DHAR), ascorbate peroxidase (APX), and polyphenoloxidase (PPO). Lowest enzyme activities, indicative of high stress and minimal defense against oxidative damage, corresponded to the CK recording GR (0.68 nmol NADPH/min), DHAR (0.54 nmol dehydroascorbate/min/g FW), APX (7.89 µmol ascorbate/min), and PPO (1.47 U/mL) (Fig. 4). Incremental increases in enzyme activities with higher concentrations of PGPR. PGPR3 showed the highest activities among individual PGPR treatments, recording GR (0.85 nmol NADPH/min), DHAR (0.90 nmol dehydroascorbate/min/g FW), APX (12.28 µmol ascorbate/min), and PPO (1.80 U/mL). Both 5 and 10 ton/ha BC alone improved enzyme activities compared to the CK. The 10% BC resulted in higher activities than 5 ton/ha BC, recording GR (1.02 nmol NADPH/min), DHAR (1.10 nmol dehydroascorbate/min/g FW), APX (14.65 µmol ascorbate/min), and PPO (1.82 U/mL). The combination of BC and PGPR yielded the highest enzyme activities, indicating a synergistic effect. The 10BC+PGPR3 (best treatment overall) had activity of GR 1.25 (nmol NADPH/min), DHAR 1.37 (nmol dehydroascorbate/min/g FW), APX (17.52 µmol ascorbate/min), and PPO (2.06 U/mL). The 10% BC+PGPR3 combination demonstrated the highest antioxidant enzyme activities across all measured parameters, making it the most effective treatment in mitigating heavy metal stress and supporting plant growth.

### Photosynthetic performance and pigment composition

**Fig. 5:**
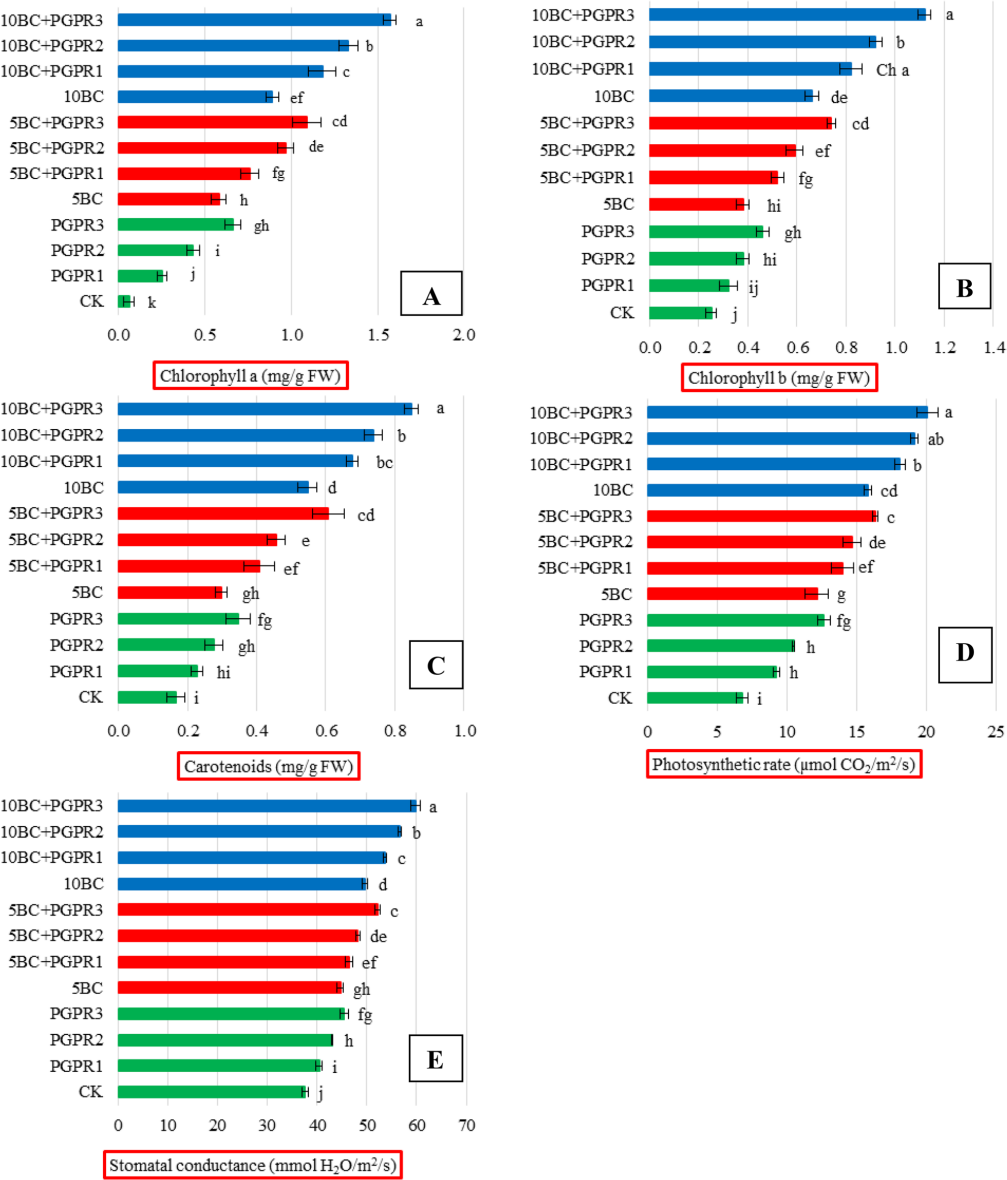
(**A**) chlorophyll a, (**B**) chlorophyll b, and (**C**) carotenoids concentrations, (**D**) photosynthetic rate, and (**E**) stomatal conductance of canola (*Brassica napus* L.) irrigated with drainage water from the Kitchener drain in contaminated soil with Pb, Cd, and Ni and treating with biochar (5 and 10 ton/ ha BC) which is fortified with three different PGPR consortia, i.e., PGPR1 (*Bacillus circulans* NCAIM B.02324 + *Azospirillum brasiliense* SARS 1001), PGPR2 (*Pseudomonas koreensis* MG209738 + *Azospirillum brasiliense* SARS 1001), and PGPR3 (*Bacillus circulans* NCAIM B.02324 + *Azospirillum brasiliense* SARS 1001 + *Pseudomonas koreensis* MG209738). A negative control was applied (CK). Different letters on bars are significant according to the Tukey’s test (*p* ≤ 0.05). Data are means ± SD (standard deviation) (*n* = 4)

Contents (mg/g FW) of chlorophyll a, chlorophyll b, and carotenoids were significantly enhanced with the addition of BC and PGPR (Fig. 5). The highest values were observed in the 10BC+PGPR3 treatment, with chlorophyll a of 1.57, chlorophyll b of 1.12, and carotenoids of 0.85. Both photosynthetic rate (µmol CO_2_/m^2^/s) and stomatal conductance (mmol H_2_O/m^2^/s) improved markedly with the treatments, especially with the combination treatments. The 10BC+PGPR3 treatment exhibited the highest photosynthetic rate (20.1) and stomatal conductance (59.9). The combination of BC and PGPR consistently outperformed individual treatments in enhancing biochemical and photosynthesis-related parameters. This suggests a synergistic effect between BC and PGPR in mitigating heavy metal stress and promoting plant growth. The 10BC+PGPR3 treatment was the most effective, significantly improving all measured parameters. BC alone at 10 ton/ha was effective in improving biochemical and photosynthesis-related parameters compared to the CK. However, its effects were less pronounced compared to the combination treatments. Among the PGPR treatments, PGPR3 was the most effective, showing better performance in improving all parameters compared to PGPR1 and PGPR2.

### Ion homeostasis (Na⁺ and K⁺ balance)

**Fig. 6:**
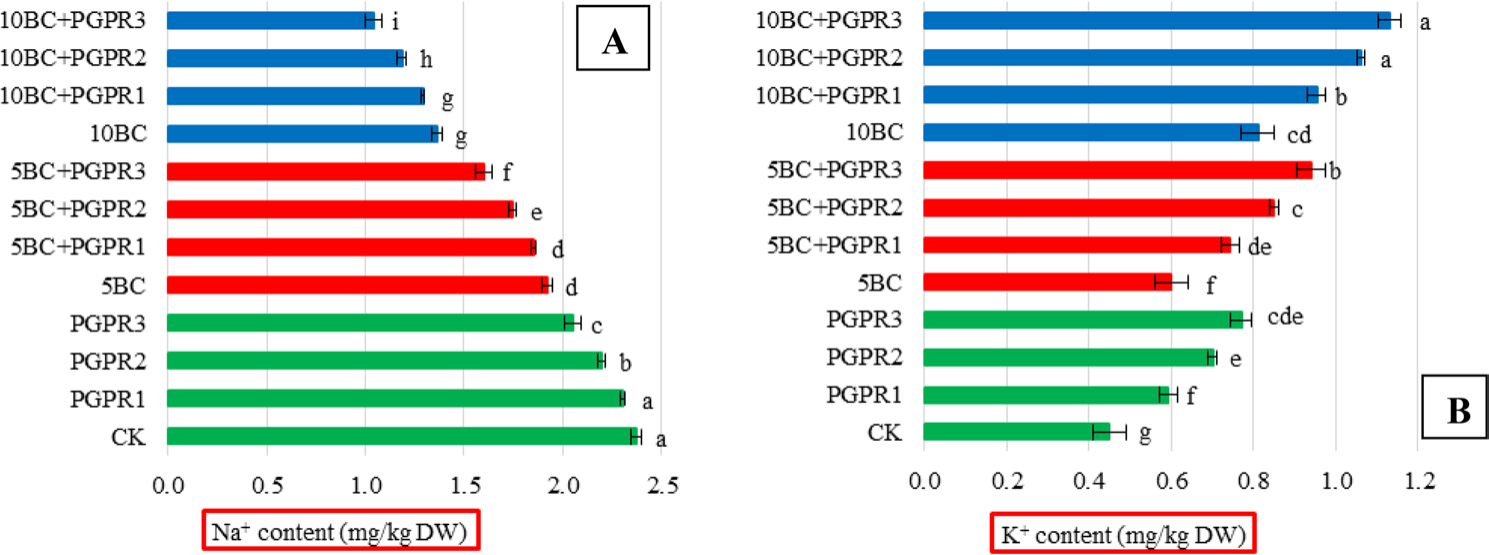
(**A**) Na^+^ and (**B**) K^+^ contents in leaves of canola (*Brassica napus* L.) irrigated with drainage water from the Kitchener drain in contaminated soil with Pb, Cd, and Ni and treating with biochar (5 and 10 ton/ ha BC) which is fortified with three different PGPR consortia, i.e., PGPR1 (*Bacillus circulans* NCAIM B.02324 + *Azospirillum brasiliense* SARS 1001), PGPR2 (*Pseudomonas koreensis* MG209738 + *Azospirillum brasiliense* SARS 1001), and PGPR3 (*Bacillus circulans* NCAIM B.02324 + *Azospirillum brasiliense* SARS 1001 + *Pseudomonas koreensis* MG209738). A negative control was applied (CK). Different letters on bars are significant according to the Tukey’s test (*p* ≤ 0.05). Data are means ± SD (standard deviation) (*n* = 4)

Figure 6 summarizes the effects of different treatments on the Na^+^ and K^+^ contents in canola plants grown in heavy metal contaminated soil. Na^+^ content decreased with the application of BC and PGPR. The combination treatments (BC+PGPR) showed the highest significant reduction. The lowest Na^+^ content (1.04) corresponded to the 10BC+PGPR3 treatment. K^+^ content increased with the treatments, particularly when BC and PGPR were combined. The highest K^+^ content (1.13) corresponded to the 10BC+PGPR3 treatment. The combination of BC and PGPR consistently outperformed individual treatments in enhancing K^+^ content while reducing Na^+^ content. This suggests a synergistic effect between BC and PGPR in mitigating heavy metal stress and promoting plant growth. The 10BC+PGPR3 treatment emerged as the best, significantly improving all measured parameters. BC alone at 10 ton/ha was effective in reducing Na^+^ content and increasing K^+^ content compared to the CK.

### Variations of heavy metals accumulation in plant tissues

**Fig. 7:**
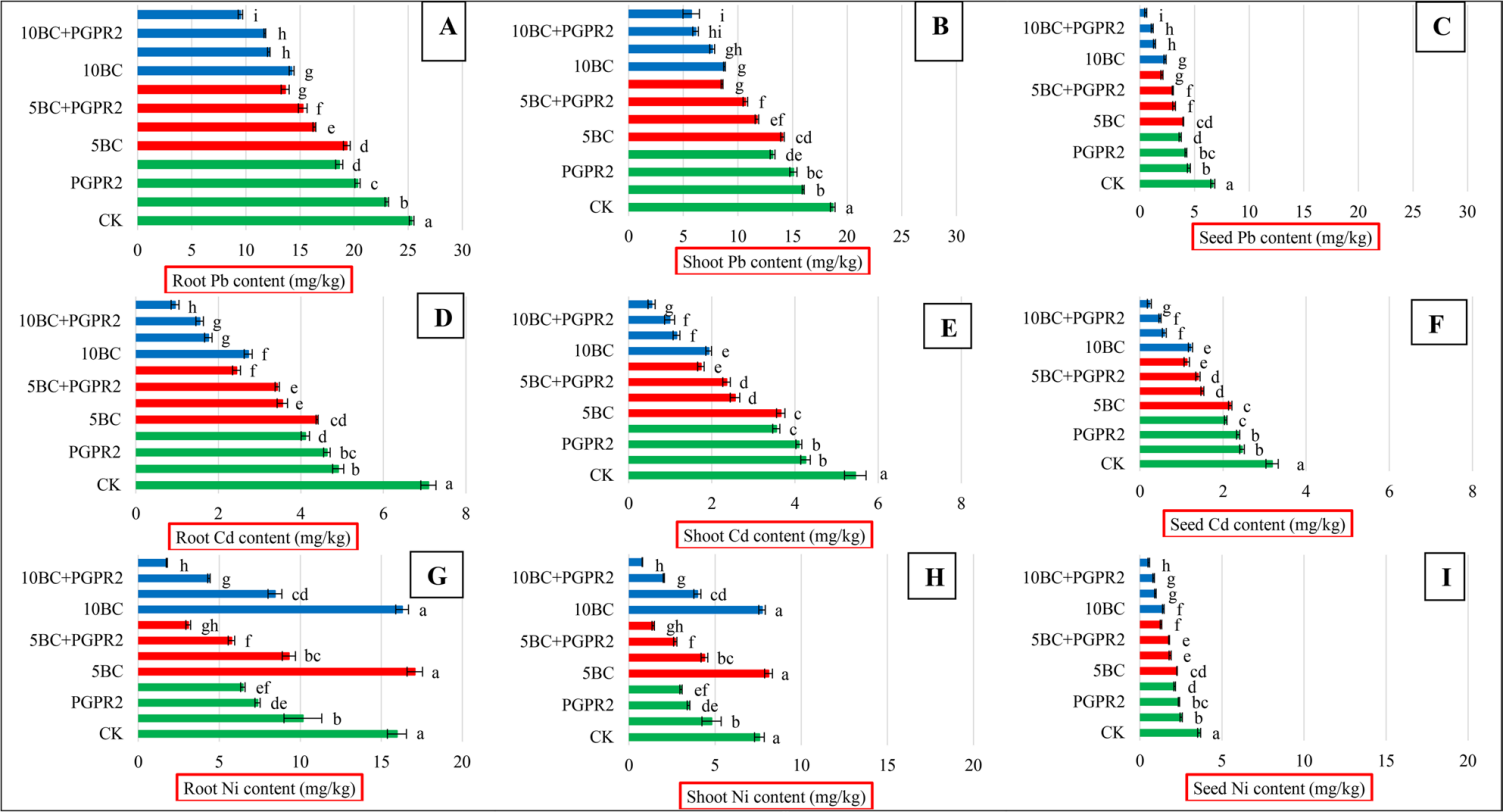
Contents of Pb (**A**, **B**, and **C**), Cd (**D**, **E**, and **F**), and Ni (**G**, **H**, and **I**) in root, shoot, and seeds, respectively, of canola (Brassica napus L.) irrigated with drainage water from the Kitchener drain in contaminated soil with Pb, Cd, and Ni and treating with biochar (5 and 10 ton/ ha BC) which is fortified with three different PGPR consortia, i.e., PGPR1 (Bacillus circulans NCAIM B.02324 + Azospirillum brasiliense SARS 1001), PGPR2 (Pseudomonas koreensis MG209738 + Azospirillum brasiliense SARS 1001), and PGPR3 (Bacillus circulans NCAIM B.02324 + Azospirillum brasiliense SARS 1001 + Pseudomonas koreensis MG209738). A negative control was applied (CK). Different letters on bars are significant according to the Tukey’s test (*p* ≤ 0.05). Data are means ± SD (standard deviation) (*n* = 4)

The combination treatments, particularly 10BC+PGPR3, resulted in the most significant reduction of heavy metal content (µg/g) in the roots, shoots, and seeds of the plants (Fig. 7). This treatment combination led to a notable decrease in Pb (root: 9.50, shoot: 5.72, seed: 0.53), Cd (root: 0.95, shoot: 0.55, seed: 0.23), and Ni (root: 1.76, shoot: 0.78, seed: 0.54) compared to the CK, which showed the highest levels of these metals.

### Heavy metal uptake and translocation indices

**Fig. 8:**
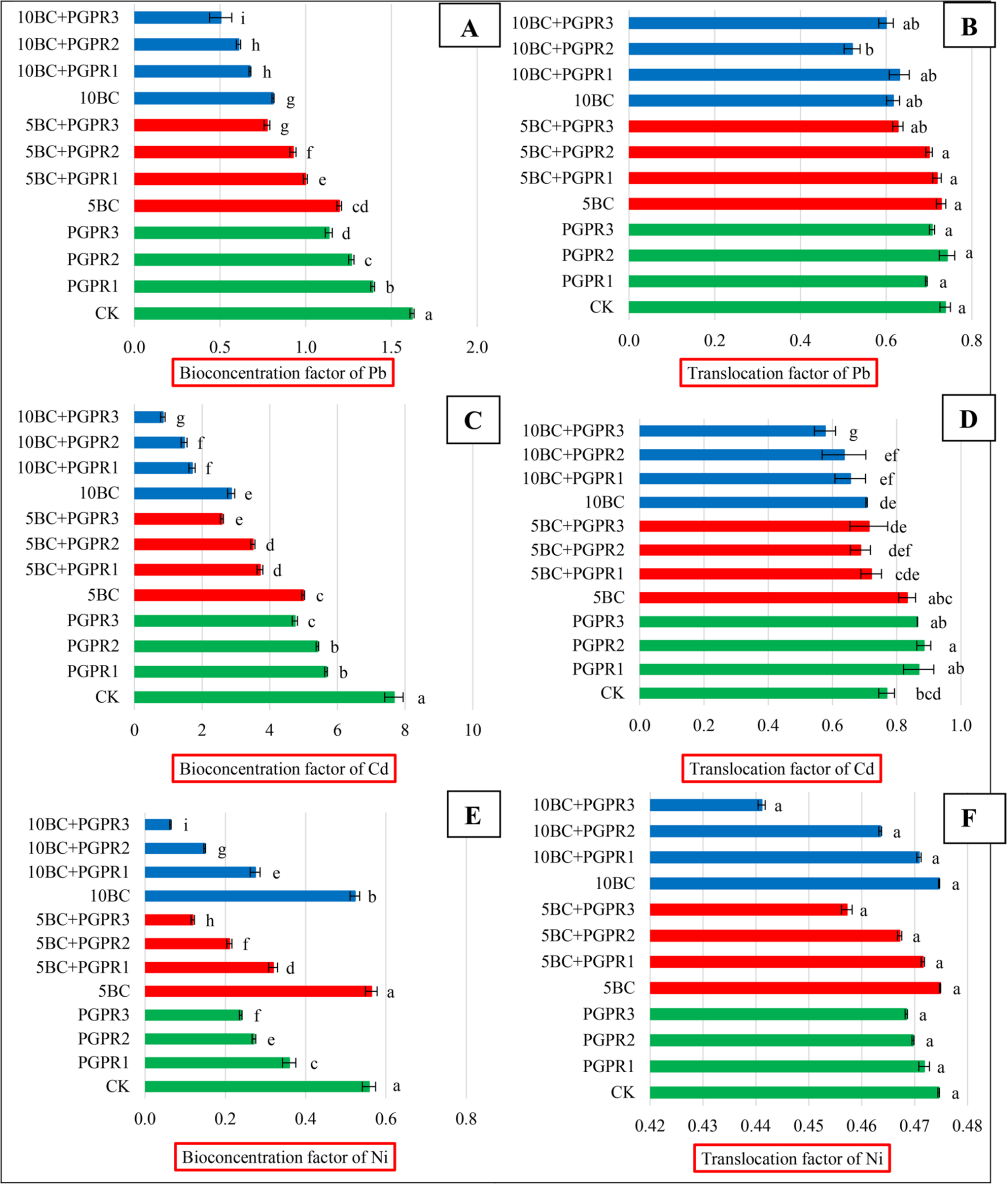
Bioconcentration (BCF) and translocation factor (TF) of Pb (**A** and **B**), Cd (**C** and **D**), and Ni (**E** and **F**) in canola (*Brassica napus* L.) irrigated with drainage water from the Kitchener drain in contaminated soil with Pb, Cd, and Ni and treating with biochar (5 and 10 ton/ ha BC) which is fortified with three different PGPR consortia, i.e., PGPR1 (*Bacillus circulans* NCAIM B.02324 + *Azospirillum brasiliense* SARS 1001), PGPR2 (*Pseudomonas koreensis* MG209738 + *Azospirillum brasiliense* SARS 1001), and PGPR3 (*Bacillus circulans* NCAIM B.02324 + *Azospirillum brasiliense* SARS 1001 + *Pseudomonas koreensis* MG209738). A negative control was applied (CK). Different letters on bars are significant according to the Tukey’s test (*p* ≤ 0.05). Data are means ± SD (standard deviation) (*n* = 4)

The combined treatment of 10BC+PGPR3 was the most effective, resulting in the lowest concentrations of Pb, Cd, and Ni in plant tissues, with seed Pb, Cd, and Ni contents reduced to 0.53 µg/g, 0.23 µg/g, and 0.54 µg/g, respectively (Fig. 8). Additionally, the BCF and TF values showed significant reductions, indicating lower trace metal phytoavailability and transportation within the plants. For instance, the BCF for Pb, Cd, and Ni under this treatment dropped to 0.50, 0.84, and 0.06, respectively, and the TF values also decreased substantially.

### Seed and oil yields of canola

Data presented in Table 1 show that the treatment of 10BC+PGPR3 was the most effective. This treatment resulted in the highest plant height (133 cm), number of pods (13.51), number of branches (7.29), 1000-seed weight (5.44 g), seed yield (3246 kg/ha), and oil content (47.1%). Comparatively, the CK treatment exhibited the lowest values across all parameters, highlighting the severe impact of heavy metal stress on plant growth and productivity. The combined BC and PGPR treatments consistently outperformed singular applications, with 10BC+PGPR3 showing the most pronounced benefits, likely due to the synergistic effects that enhance nutrient availability and reduce heavy metal toxicity. Therefore, integrating BC and PGPR, especially at higher BC concentrations, is a promising strategy for improving crop performance in heavy metal-contaminated soils.

**Table 1:** Yield and yield-related traits of canola (Brassica napus L.) irrigated with drainage water from the Kitchener drain in contaminated soil with Pb, Cd, and Ni and treating with biochar (5 and 10 ton/ ha BC), which is fortified with three different PGPR consortia, i.e., PGPR1 (Bacillus circulans NCAIM B.02324 + Azospirillum brasiliense SARS 1001), PGPR2 (Pseudomonas koreensis MG209738 + Azospirillum brasiliense SARS 1001), and PGPR3 (Bacillus circulans NCAIM B.02324 + Azospirillum brasiliense SARS 1001 + Pseudomonas koreensis MG209738). A negative control was applied (CK). Different letters on bars are significant according to the Tukey’s test (*p* ≤ 0.05)

	Plant height (cm)	No. of pods	No. of branches	1000-Seed weight (g)	Seed yield (kg/ ha)	Oil percent (%)
CK	109 ± 0.05 i	7.93 ± 0.40 h	4.30 ± 0.09 i	2.45 ± 0.10i	2039 ± 34 i	24.8 ± 0.3 g
PGPR1	113 ± 0.93 h	8.78 ± 0.19 gh	4.85 ± 0.05 h	3.00 ± 0.06 h	2241 ± 19 h	29.8 ± 0.9 f
PGPR2	117 ± 0.65 g	9.41 ± 0.29 fg	5.13 ± 0.02 h	3.28 ± 0.03 h	2238 ± 32 h	30.3 ± 0.3 ef
PGPR3	122 ± 0.36 e	9.69 ± 0.43 ef	5.62 ± 0.10 fg	3.77 ± 0.09 fg	2495 ± 49 f	34.1 ± 1.1 de
5BC	119 ± 0.85 f	9.62 ± 0.27 efg	5.51 ± 0.19 g	3.66 ± 0.18 g	2380 ± 40 g	32.3 ± 0.6 def
5BC+PGPR1	124 ± 0.11 d	10.44 ± 0.24 de	5.92 ± 0.18 ef	4.07 ± 0.19 ef	2665 ± 31 e	36.4 ± 1.3 cd
5BC+PGPR2	126 ± 0.71 c	10.96 ± 0.20 cd	6.08 ± 0.14 de	4.23 ± 0.14 de	2687 ± 49 de	39.1 ± 0.8 c
5BC+PGPR3	128 ± 0.78 c	11.46 ± 0.18 c	6.45 ± 0.04 c	4.60 ± 0.04 c	2907 ± 41 c	44.5 ± 1.2 ab
10BC	127 ± 0.70 c	11.32 ± 0.17 cd	6.33 ± 0.06 cd	4.48 ± 0.06 cd	2786 ± 32 d	40.5 ± 0.3 bc
10BC+PGPR1	130 ± 0.38 b	12.43 ± 0.64 b	6.85 ± 0.08 b	5.00 ± 0.08 b	3048 ± 16 b	45.0 ± 0.5 a
10BC+PGPR2	131 ± 0.30 b	12.93 ± 0.08ab	7.08 ± 0.07 ab	5.23 ± 0.07 ab	3055 ± 17 b	44.5 ± 0.1 ab
10BC+PGPR3	133 ± 0.07 a	13.51 ± 0.15 a	7.29 ± 0.17 a	5.44 ± 0.17 a	3246 ± 22 a	47.1 ± 0.2 a

### Pearson correlation

**Fig. 9:**
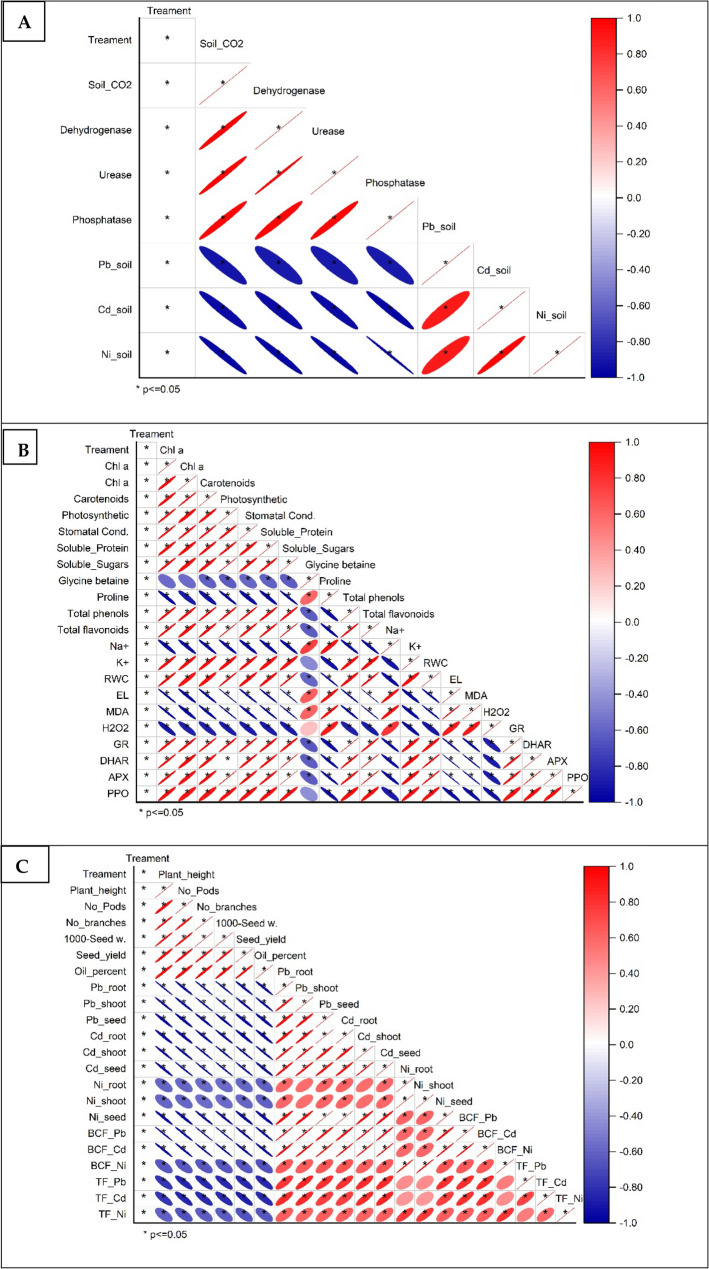
Pearson correlation coefficient of: (**A**) soil parameters, (**B**) photosynthetic pigments, non-enzymatic, enzymatic, and osmolytes, and (**C**) yield, yield-components, and yield quality of canola plants after irrigation with drainage water from the Kitchener drain in contaminated soil with Pb, Cd, and Ni and treating with biochar (5 and 10 ton/ ha) which is fortified with three different PGPR consortia, i.e., PGPR1 (*Bacillus circulans* NCAIM B.02324 + *Azospirillum brasiliense* SARS 1001), PGPR2 (*Pseudomonas koreensis* MG209738 + *Azospirillum brasiliense* SARS 1001), and PGPR3 (*Bacillus circulans* NCAIM B.02324 + *Azospirillum brasiliense* SARS 1001 + *Pseudomonas koreensis* MG209738)

The Pearson correlation supports the aforementioned findings regarding the beneficial effects of BC and PGPR on canola development and productivity, as well as food safety under heavy metal contamination conditions (Fig. 9). The proposed treatments exhibited significant and strong correlations (> 0.67) with soil respiration, activities of soil dehydrogenase, urease, and phosphatase, chlorophyll a, chlorophyll b, carotenoids, photosynthetic rate, stomatal conductance, soluble protein, TSS, TPC, TFC, K^+^, RWC, antioxidant enzyme activities (GR, DHAR, APX, and PPO), plant height, number of pods, number of branches, 1000-seed weight, seed yield, and oil percentage, highlighting improvements in soil and plant health due to BC and PGPR use. Conversely, stress indicators such as extractable Pb, Cd, and Ni contents, GB, proline, Na^+^, EL, MDA, H_2_O_2_, and Pb, Cd, and Ni accumulation in various plant organs (root, shoot, and seed) showed significant negative correlations (< 0.33) with the treatments (BC and PGPR). This further demonstrates the synergistic effect of BC and PGPR on canola growth and productivity in heavy metal-contaminated environments.

**Fig. 10:**
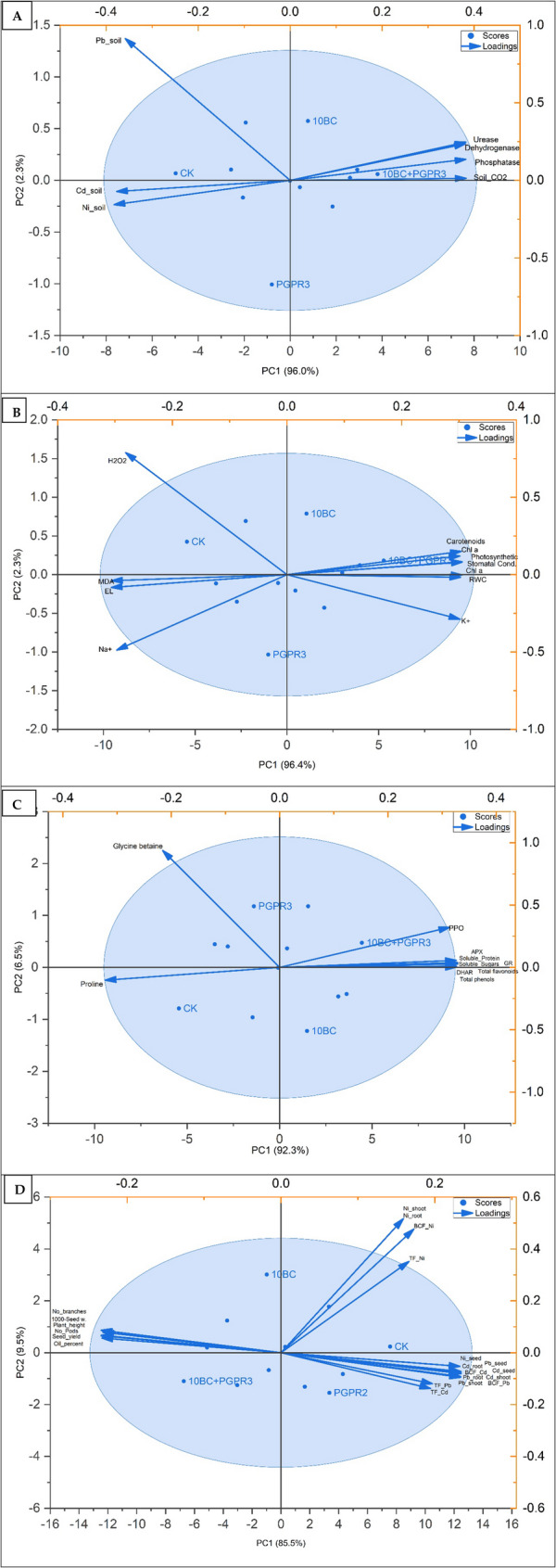
Principal component analysis (PCA) of individual soil response variables (**A**) and canola traits (**B**, **C**d, and **D**) assessed in canola plants irrigated with drainage water from the Kitchener drain in contaminated soil with Pb, Cd, and Ni after treating with biochar (5 and 10 ton/ ha) which is fortified with three different PGPR consortia, i.e., PGPR1 (*Bacillus circulans* NCAIM B.02324 + *Azospirillum brasiliense* SARS 1001), PGPR2 (*Pseudomonas koreensis* MG209738 + *Azospirillum brasiliense* SARS 1001), and PGPR3 (*Bacillus circulans* NCAIM B.02324 + *Azospirillum brasiliense* SARS 1001 + *Pseudomonas koreensis* MG209738)

The PCA plot clearly indicates that the combined treatment of BC and PGPR had the most pronounced positive effect on plant growth and yield (Fig. 10). This treatment cluster is associated with higher levels of chlorophyll a, chlorophyll b, and carotenoids, enhanced physiological parameters (stomatal conductance, soluble protein, and TSS), and improved stress tolerance markers (proline, GB, TPC, and TFC). Additionally, the combined treatment effectively reduced oxidative stress indicators such as EL, MDA, and H_2_O_2_. These results align well with recent studies which have shown that the integration of BC and PGPR can significantly ameliorate heavy metal stress in plants by improving nutrient uptake, enhancing microbial activity, and reducing metal bioavailability (Aamer et al., 2023; Gupta et al., 2022). Consequently, this combined treatment strategy demonstrates substantial potential for improving the cultivation of canola in contaminated soils.

## Discussion

Relying on the Kitchener drain to irrigate agricultural crops in northern Egypt poses significant risks of food contamination due to the accumulation of inorganic pollutants, particularly heavy metals like Pb, Cd, and Ni. This contamination arises because the drain receives wastewater from agricultural, domestic, and industrial sources in varying proportions, as confirmed by soil and plant analysis results from this study (Figs. 1 and 7). Repeated use of water from the Kitchener drain increases the soil’s heavy metal load, as noted in previous studies [15, 56]. This contamination creates a substantial threat to plant growth due to the enhanced mobility of these metals in the soil profile and the increased concentrations of their phytoavailable forms [57]. However, the mobility and bioavailability of Pb, Cd, and Ni in soil are influenced by various factors, including soil pH, organic matter content, clay composition, cation exchange capacity, redox potential, competing ions, soil texture, moisture, temperature, and biological activity [58].

High concentrations of Pb, Cd, and Ni in plant tissues can disrupt chloroplast structure and interfere with chlorophyll synthesis, impairing photosynthetic function [15, 59, 60]. Additionally, these metals induce ROS production in plant cells, causing oxidative stress that damages lipids, proteins, and nucleic acids. This stress reduces the stability of chlorophyll and other pigments, further impairing photosynthesis [61]. Heavy metals can also compete with essential nutrients, such as magnesium (Mg²⁺), iron (Fe²⁺), and zinc (Zn²⁺), critical for chlorophyll synthesis and function. Their deficiency leads to reduced pigment content and photosynthetic efficiency [62]. Furthermore, heavy metals interfere with stomatal regulation, reducing CO₂ uptake and impairing water regulation, negatively impacting photosynthesis [63]. These mechanisms were evident in this study, where increased bioavailable Pb, Cd, and Ni in soil (Fig. 1) led to higher concentrations of these metals in plant tissues, resulting in reduced photosynthetic rate, stomatal conductance, and photosynthetic pigment content (Fig. 5). Elevated metal levels also increased ROS levels, lipid peroxidation, electrolyte leakage (EL), and proline content while decreasing relative water content (RWC), soluble proteins, total soluble sugars (TSS), total phenolic content (TPC), total flavonoid content (TFC), and potassium (K⁺) levels (Figs. 2, 3 and 4). These physiological disruptions led to reduced plant height, branching, seed yield, and oil content (Table 1). Similar findings have been reported in wheat and rice, where Cd exposure reduced plant height due to protein denaturation, impairing growth and productivity [64, 65].

Both BC and PGPR play significant roles in mitigating Pb, Cd, and Ni stress in soil and water systems [15, 66]. The porous structure and high surface area of BC facilitate the immobilization of heavy metals by adsorbing Pb, Cd, and Ni, thus reducing their bioavailability and plant uptake [15, 65]. In this study, BC application at 10 tons/ha significantly decreased extractable Pb, Cd, and Ni concentrations in the soil. Similar reductions have been observed in other studies, where BC amendment reduced heavy metal bioavailability by enabling complexation with functional groups on BC surfaces and physical adsorption within its porous matrix [14, 67]. PGPR also contributes to heavy metal mitigation by producing siderophores, organic acids, and exopolysaccharides that bind heavy metals, reducing their solubility and mobility [68]. The substantial reduction in extractable Pb, Cd, and Ni in PGPR-treated soils observed in this study aligns with this mechanism, with PGPR3 showing the highest efficacy in producing metal-binding compounds.

The application of BC and PGPR, individually and in combination, significantly influenced soil enzyme activities, including dehydrogenase, urease, and phosphatase, with notable variations across treatments (Fig. 1). The control, without BC or PGPR, exhibited the lowest enzyme activities (dehydrogenase: 13.9 µg TPF/g dry soil/24 h; urease: 37.3 mg NH_4_^+^/g dry soil/day; phosphatase: 0.59 mg phenol/g dry soil/2 h), reflecting suppressed microbial activity due to high levels of bioavailable Pb (5.91 mg/kg), Cd (0.73 mg/kg), and Ni (8.52 mg/kg) in the soil. Heavy metals are known to inhibit enzyme activities by binding to active sites or disrupting microbial cell functions, as reported by [1, 69]. In contrast, PGPR treatments alone (PGPR1, PGPR2, PGPR3) enhanced enzyme activities, with PGPR3 showing the highest values among them (dehydrogenase: 21.1 µg TPF/g dry soil/24 h; urease: 87.1 mg NH_4_^+^/g dry soil/day; phosphatase: 0.84 mg phenol/g dry soil/2 h). This improvement is attributed to PGPR’s ability to stimulate microbial communities through the production of growth-promoting substances, such as phytohormones and organic acids, which enhance microbial proliferation and metabolic activity [70, 71]. The superior performance of PGPR3 may be attributed, at least in part, to functional complementarity among its three bacterial members rather than to a previously reported use of this exact consortium. Such complementarity is plausible because PGPR consortia can combine several beneficial traits, including nitrogen fixation and phytohormone production by *Azospirillum* [72, 73], mineral solubilization and extracellular enzyme activity by *Bacillus*, and siderophore/EPS-mediated metal binding and stress mitigation by *Pseudomonas* and other PGPR [74–76]. These mechanisms can reduce heavy-metal toxicity, improve nutrient availability, and enhance plant growth in contaminated soils [74–76].

Biochar alone, applied at 5 and 10 tons/ha, also significantly increased soil enzyme activities compared to the CK, with 10BC showing greater enhancement (dehydrogenase: 24.8 µg TPF/g dry soil/24 h; urease: 112.4 mg NH_4_^+^/g dry soil/day; phosphatase: 0.92 mg phenol/g dry soil/2 h). Biochar’s high surface area and porous structure provide a habitat for microbial communities, protecting them from heavy metal toxicity and improving soil physicochemical properties, such as water retention and nutrient availability [67, 77]. These conditions favor microbial growth and enzyme production, as evidenced by the higher activities compared to the CK. The 10BC treatment outperformed 5BC, likely due to its greater capacity to adsorb heavy metals (reducing extractable Pb, Cd, and Ni by 28.7%, 20.5%, and 33.4%, respectively) and provide more surface area for microbial colonization [78]. However, the most significant increases in enzyme activities were observed in the combined BC and PGPR treatments, with 10BC+PGPR3 yielding the highest values (dehydrogenase: 37.1 µg TPF/g dry soil/24 h; urease: 187.5 mg NH_4_^+^/g dry soil/day; phosphatase: 1.12 mg phenol/g dry soil/2 h). This synergistic effect can be explained by biochar’s role as a carrier that enhances PGPR survival and activity in the rhizosphere, coupled with PGPR’s production of enzymes and metal-binding compounds that further detoxify heavy metals [15, 67]. For instance, *Bacillus circulans* and *Pseudomonas koreensis* are known to produce urease and phosphatase, which facilitate nitrogen and phosphorus cycling, respectively, while *Azospirillum brasiliense* enhances microbial activity through nitrogen fixation and phytohormone production [68, 70].

The variation in enzyme activities among treatments highlights the differential impacts of BC and PGPR. Dehydrogenase activity, a key indicator of overall microbial metabolism, showed the greatest increase in 10BC+PGPR3 (166.9% higher than CK), reflecting a robust microbial ecosystem driven by the combined amendments. Urease activity, critical for nitrogen mineralization, increased most dramatically in 10BC+PGPR3 (402.7% higher than CK), likely due to PGPR’s urease production and biochar’s ability to retain ammonium ions, reducing their loss and enhancing microbial access [70]. Phosphatase activity, essential for phosphorus solubilization, also peaked in 10BC+PGPR3 (89.8% higher than CK), supported by PGPR’s secretion of phosphatases and biochar’s enhancement of soil organic matter stability [77]. The combined treatment’s superior performance is further evidenced by the strong positive correlation between enzyme activities and soil respiration (*r* > 0.67, Fig. 9), indicating that enhanced microbial activity contributes to improved soil health and reduced heavy metal bioavailability. These findings align with Alshaal et al. [15], who reported similar synergistic effects of BC and PGPR on enzyme activities in Cd-contaminated soils under sunflower cultivation, and Zhang et al. [71], who noted enhanced microbial functions in heavy metal-stressed environments. The variations in enzyme activities underscore the potential of 10BC+PGPR3 as a sustainable strategy for improving soil health and supporting canola growth in contaminated environments by enhancing nutrient cycling and mitigating heavy metal toxicity.

Heavy metal stress induces oxidative damage in plants, leading to the accumulation of ROS such as H_2_O_2_ and MDA [61]. The study revealed that BC and PGPR treatments enhanced the antioxidant defense system of canola plants. Antioxidant enzyme activities, including GR, DHAR, APX, and PPO, were significantly increased, particularly in the 10BC+PGPR3 treatment. These enzymes play crucial roles in detoxifying ROS, thereby protecting plant cells from oxidative damage. The reduction in H_2_O_2_ and MDA content in treated plants indicates effective mitigation of oxidative stress. The combined treatment’s superior performance suggests a synergistic interaction where BC’s nutrient supply and metal immobilization, coupled with PGPR’s production of phytohormones and growth-promoting substances, collectively enhance the plant’s antioxidative capacity .

The study’s findings underscore the positive impact of BC and PGPR on soil health. Enhanced soil respiration, as observed with BC and PGPR treatments, indicates increased microbial activity and improved soil organic matter decomposition. Soil respiration was highest in the 10BC+PGPR3 treatment, reflecting a robust microbial ecosystem supported by the combined amendments. Moreover, the reduction in soil heavy metal content and the improvement in soil enzyme activities contribute to a healthier soil environment. These improvements facilitate better root growth and nutrient uptake, further enhancing plant resilience to heavy metal stress [15, 65].

Physiological health indicators, such as RWC, EL, and osmolyte accumulation, were significantly improved with BC and PGPR treatments [61]. The highest RWC and lowest EL were recorded in the 10BC+PGPR3 treatment, indicating better water retention and membrane stability under heavy metal stress. The accumulation of osmolytes like proline, soluble proteins, and TSS is a common plant response to stress. The study found that BC and PGPR treatments increased soluble protein and TSS levels while reducing proline content, particularly in the combined treatment. These changes suggest enhanced stress tolerance and metabolic activity, as osmolytes help in osmotic adjustment, protection of cellular structures, and scavenging of ROS [79].

The study demonstrated significant improvements in yield traits of canola plants treated with BC and PGPR. The combined 10BC+PGPR3 treatment resulted in the highest seed yield, oil content, and overall productivity. These improvements are likely due to enhanced nutrient availability, improved soil health, and reduced heavy metal toxicity [65]. The positive effects on yield can be attributed to several factors: increased photosynthetic rate and stomatal conductance, higher chlorophyll and carotenoid content, and improved plant physiological and biochemical health. The synergistic effect of BC and PGPR is evident, as the combined treatment consistently outperformed individual applications in promoting growth and productivity [67]. Alshaal et al. [15] demonstrated enhanced biomass and yield in sunflowers treated with both BC and PGPR in Cd contaminated soils, corroborating our observation that 10BC+PGPR3 was the most effective treatment.

Food safety is a critical concern in heavy metal-contaminated environments. The study highlighted the significant reduction in heavy metal accumulation in canola plant tissues, particularly seeds, with BC and PGPR treatments. The 10BC+PGPR3 treatment showed the lowest concentrations of Pb, Cd, and Ni in seeds, ensuring safer consumption. The reduction in BCF and TF values indicates that BC and PGPR effectively limit heavy metal uptake and translocation within the plant. This immobilization of metals in the root zone prevents their accumulation in edible parts, thereby enhancing the food safety of canola crops [80].

The superior efficacy of PGPR-enriched biochar (BC + PGPR) in reducing heavy metal uptake, particularly in the 10BC+PGPR3 treatment, compared to biochar alone, can be attributed to the synergistic interactions between biochar’s physicochemical properties and PGPR’s biological activities. While biochar alone effectively immobilizes heavy metals (Pb, Cd, and Ni) through adsorption and complexation due to its high surface area, porous structure, and functional groups (e.g., carboxyl, hydroxyl) [14, 78], the addition of PGPR enhances these processes through multiple biological mechanisms. PGPR, specifically strains like *Bacillus circulans* NCAIM B.02324, *Azospirillum brasiliense* SARS 1001, and *Pseudomonas koreensis* MG209738 used in PGPR3, are known to produce root exudates such as organic acids (e.g., citric, malic, and oxalic acids), siderophores, and extracellular polymeric substances (EPS) [68, 81]. These exudates play a critical role in altering metal bioavailability by chelating heavy metals in the rhizosphere, forming stable complexes that reduce their solubility and phytoavailability [70]. For instance, siderophores produced by *Pseudomonas* species can bind metals like Cd and Ni, decreasing their free ionic forms in the soil solution, as supported by [81]. Additionally, PGPR-induced root exudates enhance root growth and exudation rates, increasing the release of low-molecular-weight organic compounds that further bind heavy metals, limiting their uptake into plant tissues [71, 82].

Soil microbial interactions are also significantly enhanced by the combined application of BC and PGPR. Biochar provides a favorable microenvironment for microbial proliferation by improving soil structure, water retention, and nutrient availability, which supports the colonization and activity of PGPR [67, 81]. The porous structure of biochar serves as a habitat for PGPR, protecting them from environmental stresses and enabling sustained production of metal-binding compounds. In this study, the 10BC+PGPR3 treatment exhibited the highest soil microbial activity, as evidenced by elevated soil respiration (44.6 mg CO_2_/100 g dry soil/24 h) and enzyme activities (dehydrogenase: 37.1 µg TPF/g dry soil/24 h; urease: 187.5 mg NH_4_^+^/g dry soil/day; phosphatase: 1.12 mg phenol/g dry soil/2 h) (Fig. 1). These enhanced microbial activities likely result from PGPR’s ability to stimulate native soil microbial communities, which produce enzymes such as urease and phosphatase that facilitate nutrient cycling and indirectly reduce metal bioavailability by stabilizing soil organic matter [70]. For example, *Bacillus circulans* and *Azospirillum brasiliense* are known to secrete enzymes that degrade organic matter, releasing organic ligands that complex with heavy metals, further immobilizing them in the soil matrix [68].

Enzymatic processes induced by PGPR also contribute to the reduced bioavailability of heavy metals. PGPR strains produce enzymes such as metallothioneins and phytochelatins, which bind heavy metals in the rhizosphere, preventing their uptake by plant roots [70, 71]. Additionally, PGPR enhance the activity of soil enzymes like dehydrogenase, which is a key indicator of microbial metabolic activity and plays a role in redox reactions that transform heavy metals into less bioavailable forms [69]. The synergistic effect of BC and PGPR is evident in the 10BC+PGPR3 treatment, which not only reduced extractable Pb, Cd, and Ni concentrations by 55.3%, 45.2%, and 72.1%, respectively, compared to the control (Fig. 1), but also minimized metal accumulation in canola seeds (Pb: 0.53 µg/g; Cd: 0.23 µg/g; Ni: 0.54 µg/g) (Fig. 7). This suggests that PGPR-induced enzymatic processes, combined with biochar’s adsorption capacity, create a robust barrier to metal translocation, as evidenced by the reduced BCF and TF values (Fig. 8). Furthermore, PGPR’s production of phytohormones (e.g., indole-3-acetic acid, gibberellins) promotes root elongation and branching, enhancing the rhizosphere’s capacity to sequester metals and improving plant vigor, which indirectly reduces metal uptake by maintaining healthier root systems [82].

In contrast, biochar alone primarily relies on its physicochemical properties to immobilize heavy metals, lacking the dynamic biological interactions provided by PGPR. While biochar at 10 tons/ha significantly reduced extractable heavy metal concentrations compared to the control, its effects were less pronounced than those of the combined 10BC+PGPR3 treatment (Fig. 1). The absence of PGPR limits the production of metal-binding exudates and enzymes, resulting in lower microbial activity and less effective metal immobilization. The combined treatment leverages biochar’s ability to provide a stable substrate for PGPR activity while enhancing soil microbial interactions and enzymatic processes, leading to a more comprehensive reduction in heavy metal bioavailability and uptake. These findings align with recent studies [15, 67], which report that the integration of biochar and PGPR enhances metal immobilization through complementary physicochemical and biological mechanisms, offering a sustainable strategy for remediating contaminated soils and improving crop safety and productivity.

The synergistic benefits of PGPR-enriched BC were evident across all measured parameters. This synergy can be attributed to several mechanisms: (1) enhanced nutrient availability—BC improves soil nutrient content while PGPR enhances nutrient uptake through root growth promotion and phytohormone production [67]; (2) improved microbial activity—BC provides a habitat for microbial communities, while PGPR boosts microbial functions, leading to a more active soil ecosystem [81]; (3) robust antioxidant defense—the combined treatment significantly enhances antioxidant enzyme activities, mitigating oxidative stress [65]; and (4) synergistic stress alleviation—BC immobilizes heavy metals, and PGPR promotes growth and stress tolerance, creating an effective mitigation strategy for heavy metal-contaminated environments [15]. This combined approach ensures higher yields and safer food production, demonstrating its potential as a sustainable agricultural practice in contaminated areas.

While the findings of this study highlight the significant potential of BC and PGPR, particularly the 10BC+PGPR3 treatment, in mitigating heavy metal stress and enhancing canola productivity, several limitations must be considered. First, the experiment was conducted over a single growing season in 2022 at a specific site in Kafr El-Sheikh, Egypt, which limits the generalizability of the results across multiple seasons. Seasonal variations in environmental factors, such as temperature, precipitation, or irrigation water quality from the Kitchener drain, could influence the efficacy of BC and PGPR treatments. Future studies should include multi-season trials to assess the consistency of these effects over time. Second, the study was performed on clayey soil with specific physicochemical properties (pH 8.20, SOM 11.1 g/kg, EC 4.64 dS/m), and the performance of BC and PGPR may vary in soils with different textures (e.g., sandy or loamy), organic matter content, or pH levels, as well as in diverse climatic zones. Further research across a range of soil types and climates is needed to confirm the broader applicability of these treatments. Third, while PGPR3 demonstrated robust plant growth-promoting and heavy metal-mitigating effects, the long-term sustainability of PGPR colonization in the rhizosphere remains uncertain [83]. Factors such as microbial competition, soil microbial diversity, and environmental stressors may affect PGPR persistence and efficacy over multiple seasons. Long-term field studies and molecular analyses of microbial communities could elucidate the dynamics of PGPR colonization and inform strategies for sustained application, such as periodic reinoculation or optimized carrier materials. Addressing these limitations through future research will enhance the understanding of BC and PGPR as sustainable solutions for heavy metal-contaminated agricultural systems.

## Conclusions

This study demonstrates that the combined application of biochar and PGPR effectively mitigates heavy metal stress in canola grown in contaminated soil. The 10 t ha⁻¹ biochar plus PGPR3 treatment (10BC+PGPR3) showed the strongest overall performance, reducing extractable soil Pb, Cd, and Ni by 55.3%, 45.2%, and 72.1%, respectively, while markedly enhancing soil enzyme activities and overall soil biological health. This treatment alleviated plant stress, as indicated by increased relative water content and reduced electrolyte leakage and lipid peroxidation. Consequently, seed yield and oil content increased by 59.2% and 89.9%, respectively, compared with the control, while heavy metal accumulation in seeds was substantially reduced, ensuring improved crop safety. These results highlight the synergistic role of biochar and PGPR in improving soil functionality, plant resilience, and productivity under heavy metal contamination. Future research should evaluate the performance of PGPR-enriched biochar across different soil types and contamination levels and assess its long-term sustainability. Multi-season field studies are particularly needed to examine the persistence of soil biological improvements, heavy metal immobilization, and the economic feasibility of large-scale application.

## Data Availability

Data is provided within the manuscript or supplementary information files.
